# A Micro RNA Processing Defect in Rapidly Progressing Idiopathic Pulmonary Fibrosis

**DOI:** 10.1371/journal.pone.0021253

**Published:** 2011-06-21

**Authors:** Sameer R. Oak, Lynne Murray, Athula Herath, Matthew Sleeman, Ian Anderson, Amrita D. Joshi, Ana Lucia Coelho, Kevin R. Flaherty, Galen B. Toews, Darryl Knight, Fernando J. Martinez, Cory M. Hogaboam

**Affiliations:** 1 Department of Pathology, University of Michigan Medical School, Ann Arbor, Michigan, United States of America; 2 MedImmune, Cambridge, United Kingdom; 3 Division of Pulmonary and Critical Care Medicine, Department of Internal Medicine, University of Michigan Health System, Ann Arbor, Michigan, United States of America; 4 Department of Anesthesiology, Pharmacology and Therapeutics, UBC James Hogg Research Centre of the Heart+Lung Institute, University of British Columbia, Vancouver, British Columbia, Canada; University of Pittsburgh, United States of America

## Abstract

**Background:**

Idiopathic pulmonary fibrosis exhibits differential progression from the time of diagnosis but the molecular basis for varying progression rates is poorly understood. The aim of the present study was to ascertain whether differential miRNA expression might provide one explanation for rapidly versus slowly progressing forms of IPF.

**Methodology and Principal Findings:**

miRNA and mRNA were isolated from surgical lung biopsies from IPF patients with a clinically documented rapid or slow course of disease over the first year after diagnosis. A quantitative PCR miRNA array containing 88 of the most abundant miRNA in the human genome was used to profile lung biopsies from 9 patients with rapidly progressing IPF, 6 patients with slowly progressing IPF, and 10 normal lung biopsies. Using this approach, 11 miRNA were significantly increased and 36 were significantly decreased in rapid biopsies compared with normal biopsies. Slowly progressive biopsies exhibited 4 significantly increased miRNA and 36 significantly decreased miRNA compared with normal lung. Among the miRNA present in IPF with validated mRNA targets were those with regulatory effects on epithelial-mesenchymal transition (EMT). Five miRNA (miR-302c, miR-423-5p, miR-210, miR-376c, and miR-185) were significantly increased in rapid compared with slow IPF lung biopsies. Additional analyses of rapid biopsies and fibroblasts grown from the same biopsies revealed that the expression of *AGO1 and AGO2* (essential components of the miRNA processing RISC complex) were lower compared with either slow or normal lung biopsies and fibroblasts.

**Conclusion:**

These findings suggest that the development and/or clinical progression of IPF might be the consequence of aberrant miRNA processing.

## Introduction

Idiopathic pulmonary fibrosis (IPF) is a progressive fibroproliferative disorder characterized by excessive, irreversible scarring of the lungs [1]. The incidence and prevalence of IPF have both steadily increased over the past two decades [2], and this disease presently claims more lives annually in the United States than many types of cancer [3]. From the time of a definitive diagnosis of IPF, patient prognosis is grim since their median survival time is approximately 2.8 years [4]. IPF is characterized by pronounced collagen deposition and other alterations to the extracellular matrix, which dramatically remodels and stiffens the lung's distal airspaces and parenchyma [3]. Difficulty breathing and eventual death are caused by incurrent pneumonia or respiratory failure. Currently, pharmacologic treatments for IPF are ineffective at halting the IPF progression and treatment options aside from lung transplantation are the focus of active investigation [5]. There is presently no consensus on the etiopathogeneis of IPF, but various genetic and environmental factors have been implicated [3].

Although a high degree of variability in IPF progression has been observed in patients [6]–[9], the identification of key indicators that predict disease progression has been elusive. Some have proposed that high-resolution computed tomography can be employed to identify IPF patients at greater risk of earlier death [10], but this diagnostic approach has recently been challenged as being unreliable [11]. Molecular analysis of lung tissue resected for diagnostic purposes have provided more encouraging results suggesting that IPF lung biopsies have a unique messenger RNA transcriptome compared with non-fibrotic or normal control biopsy samples [12], [13]. This molecular approach has been extended toward defining biologically relevant transcript differences in IPF patients with differing disease progression [7], [8], [14]–[17]. Thus, previous studies have highlighted that the analysis of molecular transcripts from IPF patients might aid in enhancing our understanding of the biological processes driving lung fibrosis, which in turn might aid in the identification of patients at greatest risk for rapid progression.

There is growing evidence that the regulation of gene transcription in health and disease involves several non-redundant mechanisms. One such mechanism involves microRNA, which are 22 nucleotide non-coding RNA molecules that exert a biologically important effect on post-transcriptional gene expression [18]. Each miRNA is predicted to target hundreds of mRNA transcripts [19] and approximately one-third of human genes are targeted or regulated via miRNA-dependent processes [20]. MiRNA biosynthesis in the cell nucleus is regulated by RNase III Drosha [21] and Exportin 5 [22]. In the cytoplasm, *Dicer1* is required for further processing [21], [23] before the miRNA duplex is loaded into the RNA induced silencing complex (RISC) [18], [24]. The RISC is made up of many proteins of which the Argonaute (AGO) proteins are the core catalytically active subunits [25], [26]. This large ribonucleoprotein complex uses base pairing interactions with the miRNA's 7 nucleotide long “seed region” to target the 3′UTR of different mRNA transcripts which inhibits their translation or causes their degradation [27]. The degradation of mRNA levels through destabilization accounts for the majority (≥84%) of the decreased protein output, so changes in mRNA levels can be used to estimate changes in protein production [28].

MicroRNAs have been implicated in cancer, heart and neurodegenerative diseases, diabetes, and inflammation [29]. Furthermore, miRNA have also been involved in many forms of tissue fibrosis [30]. For example, differences in miR-21, miR-29, miR-30, and miR-133 expression have been examined in cardiac fibrosis [31]–[33]. A connection between miRNA and fibrosis has also been shown in diabetic kidney sclerosis [34], liver fibrosis [35], [36], cystic fibrosis [37], and diabetic neuropathy [38]. More recently, the downregulation of let-7d [39], upregulation of miR-21 [40], and the downregulation of miR-29 [41] were shown to contribute to the enhanced fibrosis observed in IPF. However, it remains to be determined whether differential miRNA expression contributes to variations in the clinical progression of pulmonary fibrosis.

The aim of the present study was two fold: 1) to determine whether miRNA biosynthesis differed between IPF and normal lung tissues; and 2) to determine whether miRNA expression differed between IPF patients exhibiting varying speeds of disease progression. Herein, we report that the miRNA levels in diagnostic lung biopsies markedly differ in both rapidly progressive and slowly progressive IPF patients compared with normal individuals, albeit the large majority of the miRNA identified were significantly lower in the IPF biopsies compared with the normal biopsies. Among the miRNA present in IPF with validated mRNA targets were those with regulatory effects on epithelial-mesenchymal transition (EMT), a process of emerging importance in IPF [42]. A low-density mRNA array profile of the three biopsy groups revealed that the rapid IPF group had marked increases in several mesenchymal markers compared with the slow IPF and normal groups supporting. A comparison of biopsies from rapidly progressive patients with biopsies from slowly progressive patients revealed 5 miRNA that were significantly higher in the first group, suggesting that the miRNA profile in lung biopsy tissue might be useful in distinguishing rapidly from slowly progressing IPF. Finally, both *AGO1* and *AGO2* (the core components of the RISC) were expressed at lower levels in rapidly progressive IPF biopsies and/or fibroblasts grown from the same biopsies compared with both normal and slowly progressive IPF biopsies and fibroblasts. Together, these data suggest that the development of IPF and/or the pace of its clinical progression might be a consequence of abnormal miRNA generation and processing.

## Methods

### Patients

All surgical lung biopsies (SLBs) were obtained from the patient at the time of diagnosis of IPF. Patients were diagnosed with IPF using a multidisciplinary approach involving a clinicians, radiologists, and pathologists [43]. A University of Michigan Institutional Review Board approved this study and written informed consent was obtained from each patient. IPF patients were retrospectively grouped into a rapidly progressive group (n = 9; 2 female and 7 male; median age 66) based on the following criteria during the first year of follow-up: mortality or acute exacerbation, percent forced vital lung capacity (FVC) decrease of ≥10%, and percent diffusing capacity of carbon monoxide (DL_CO_) decrease of ≥15%. IPF patients that did not meet these criteria over the first year of follow-up were clinically diagnosed as slowly progressive (n = 6; 4 female and 2 male; median age = 61 years). Samples taken from disease-free patient autopsies were used as normal controls (n = 10; 5 female and 5 male; median age = 24 years). A University of British Columbia Institutional Review Board approved the procurement and analysis of these tissues.

### Total RNA and miRNA Isolation, and cDNA Generation

Left side upper and/or lower lobe surgical lung biopsies were stored at −80°C and thawed on ice immediately prior to RNA isolation. Approximately 1 ml of Trizol (Invitrogen Life Technologies, Carlsbad, CA) was added to each biopsy. Each biopsy was homogenized using the Tissue-Tearor (Biospec Products, Bartlesville, OK) and total RNA extraction was performed according to the manufacturer's instructions. Concentration and purity of each lung sample was determined using a NanoDrop 1000 UV-Vis photospectrometer (Thermo Scientific, Wilmington, DE). 1 µg of total RNA from each biopsy was converted into cDNA using Murine Moloney Leukemia Virus Reverse Transcriptase (Invitrogen Life Technologies, Carlsbad, CA). MiRNA was isolated and purified from 10 µg of total RNA according to manufacturer's instruction for the RT^2^ qPCR-Grade miRNA Isolation Kit (SA Biosciences, Frederick, MD). Concentration and purity were again measured after miRNA isolation using the NanoDrop 1000 photospectrometer and samples were screened for an A_260_∶A_280_ ratio greater than 1.8. Conversion of miRNA to cDNA was carried out using 100 ng of miRNA according to manufacturer's instructions for the RT^2^ miRNA First Strand Kit (SA Biosciences, Frederick, MD).

### MicroRNA Array Analysis

MicroRNA expression values for each sample were measured using a RT^2^ miRNA PCR Array Human miFinder (SA Biosciences, Frederick, MD). Plates contained 88 of the most abundantly expressed and best-characterized miRNAs found in humans as well as relevant housekeeping miRNA. Samples were analyzed by quantitative real time polymerase chain reaction (qRT-PCR) using the ABI 7500 Real Time PCR System (Applied Biosystems, Foster City, CA) following the manufacturer's protocol. A dissociation curve was run to factor out wells with nonspecific amplification and primer dimers. Samples were grouped as normal, slowly progressive, and rapidly progressive and analyzed using the PCR Array Data Analysis Excel Template with the ΔΔC_t_ method (SA Biosciences, Frederick, MD). Data were normalized using the small nuclear RNA housekeeping panel: SNORD 47, SNORD 44, and U6.

### Messenger RNA Target List Generation

MiRecords (http://mirecords.biolead.org/) [44] was used to compile a list of experimentally validated miRNA targets. This is a database of miRNA target interactions with published experimental support.

### Quantitative mRNA Analysis

Circular DNA (cDNA) made from total RNA was analyzed using quantitative real time polymerase chain reaction (qRT-PCR) using the ViiA 7 Real-Time PCR System (Applied Biosystems, Foster City, CA). Pre-mixed primers and probes were purchased from Applied Biosystems and used to detect human *DICER1, AGO1, and AGO2* using a 96 well format. 48 genes associated with the epithelial-mesenchymal transition (EMT) were analyzed using a preloaded custom TaqMan Array Microfluidic Card (Applied Biosystems, Foster City, CA). Data were normalized to an internal standard, *GAPDH* in the 96 well format and to *18s* in the card. Samples were grouped into either slowly progressive IPF or rapidly progressive IPF, and compared with normal using the ΔΔC_t_ method.

### Immunohistochemistry

Five-micron thick paraffin-embedded tissue sections were cut from formalin-fixed normal, slowly progressive, and rapidly progressive SLBs. Each tissue sections was de-parafinized and rehydrated by washing with xylene and a decreasing ethanol gradient. Sections were heated in 10 mM citric acid (pH 6.0) for 12 minutes for antigen retrieval and incubated for 30 minutes in a 10% methanol/ .4% H_2_O_2_ solution to permeablize tissue sections and block endogenous peroxidase activity. Tissue sections were additionally blocked for with 5% BSA/ 1% rabbit serum, Avidin Block (R and D Systems, Minneapolis, MN), and Biotin Block (R and D Systems, Minneapolis, MN). The sections were incubated overnight at 4°C in anti-AGO1 rabbit polyclonal antibody (4 or 20 µg/ml; BioLegend, San Diego, CA) or anti-AGO2 rabbit polyclonal antibody (4 or 20 µg/ml; ,Abcam, Cambridge, MA). In-house non-specific rabbit polyclonal IgG was used as a concentration matched negative control. Sections were incubated with biotinylated anti-rabbit secondary antibody (R&D Systems, Minneapolis, MN) for 1 hour then with HSS-HRP Streptavidin Peroxidase (R&D Systems, Minneapolis, MN) for 30 minutes. Tissue was stained with DAB chromogen, counterstained with hematoxylin, and photographed with an Olympus BX40 microscope and IP Lab Spectrum software (Signal Analytics Corp, Vienna, VA).

### Primary Pulmonary Fibroblast Culture and Treatment

To culture primary human fibroblasts, histologically normal and IPF SLBs were finely minced and the dispersed tissue pieces were placed into 150 cm^2^ cell culture flasks with media. The media consisted of DMEM (Lonza, Walkersville, MD) with 15% fetal bovine serum (Cell Generation, Fort Collins, CO), 2 mmol/L glutamine, and 1× Penicillin-Streptomycin-Amphotericin B (Lonza, Walkersville, MD). Cells lines were cultured in media at 37°C in a 7% CO_2_ incubator and were serially passaged 4 times to yield pure populations of adherent fibroblasts. All primary fibroblast cell lines from each patient group were used between passages 6 and 11. Prior to an experiment, fibroblasts from each group (n = 4 normal primary lines, n = 6 stable primary IPF lines, n = 4 rapid primary IPF lines) were plated into a six-well tissue culture plate with 5×10^5^ cells per well and activated for 4 hours with media alone. Trizol reagent was added to each well to terminate the experiment and mRNA was isolated and analyzed as described above.

### Statistical Analysis

Student's T-test or Mann-Whitney test for non-normally distributed data were used to determine statistical differences among the analyzed groups. Calculations were performed using PRISM 5.0 software for Macintosh (GraphPad Software, San Diego, CA). P≤0.05 was considered statistically significant. The statistical analysis performed on the EMT-associated genes used qRT-PCR generated dCT values following the (Ct of gene of interest (GOI) – Ct of housekeeping gene (HKG)) calculation. Data were clustered using the absolute value of correlation coefficients (distance measure) using hierarchical clustering to show the genes that are strongly related in each group (Normal, Slow, and Rapid) (R version 2.13.0, www.R-project.org). The “Ward” method, which derives spherical clusters has been used as the agglomeration algorithm for forming clusters.

## Results

### Differential miRNA expression in whole lung samples distinguished normal from IPF and rapidly from slowly progressing IPF

Previous studies have identified global alterations in mRNA transcript expression in IPF in terms of progression [14], [15] and in IPF with acute exacerbation [45]. To test the hypothesis that altered mRNA transcript levels in IPF might be a consequence of defective regulatory mechanisms, we investigated whether differences in miRNA expression might explain alterations in mRNA expression in this disease. Using a quantitative real time miRNA PCR array containing 88 of the most abundant miRNA found in the human genome to analyze small RNA isolated from SLBs, a number of significant differences in miRNA expression levels were found in rapidly progressing IPF lung samples compared with normal samples (**Table 1**). Of the 88 mature miRNA analyzed, 11 miRNA were significantly increased in rapid biopsies and 36 miRNA were significantly decreased compared with the normal lung samples. A comparison between slowly progressive IPF lung biopsies and normal lung biopsies revealed that 4 miRNA were significantly increased and 34 miRNA were significantly decreased (**Table 2**). Lastly, a comparison between slowly and rapidly progressive IPF biopsies revealed that 5 miRNA were significantly increased and 1 miRNA was significantly decreased in rapid IPF biopsies when compared to miRNA levels in slowly progressive biopsies (**Table 3**). Overall, these data highlight that miRNA levels in biopsies from rapidly and slowly progressive IPF exhibited marked differences from normal lung biopsies; most of the miRNA analyzed were significantly lower in IPF samples compared with normal samples. However, miRNA expression in surgical lung biopsy samples was different between rapidly and slowly progressing IPF biopsies suggesting that an analysis of miRNA biosynthesis and processing might reveal clues regarding the differing rates of progression in pulmonary fibrosis.

**Table 1:** List of increased and decreased miRNA in rapidly progressive IPF biopsies n = 9) when compared to normal lung samples (n = 10) with p≤0.05.

miRNA	p-value	Fold increase/decrease
miR-423-5p	0.0003	14.08
miR-155	0.0005	12.02
miR-128	0.0007	8.92
miR-374b	0.0062	7.87
miR-21	0.0325	6.75
miR-100	0.0052	6.40
miR-125b	0.0023	5.23
miR-140-3p	0.0047	3.97
miR-125a-5p	0.0330	3.94
miR-92a	0.0392	3.23
let-7c	0.0181	3.03
miR-181b	0.0379	−2.13
let-7d	0.0225	−2.42
miR-30c	0.0340	−2.80
miR-27b	0.0392	−3.16
miR-103	0.0055	−3.33
miR-30a	0.0305	−3.43
miR-424	0.0223	−3.91
miR-22	0.0091	−3.95
miR-186, miR-29a	0.0014	−4.22
miR-126	0.0115	−4.71
miR-27a	0.0215	−5.33
miR-20a	0.0094	−6.56
miR-143	0.0009	−6.69
miR-223	0.0021	−6.93
miR-17	0.0043	−7.46
miR-106b	0.0002	−7.52
miR-96	0.0111	−7.96
miR-140-5p	0.0033	−8.05
miR-15a	0.0003	−8.32
miR-30b	0.0003	−9.73
miR-130a	0.0003	−9.92
miR-222, miR-30e	0.0004	−10.54
miR-29c	0.00003	−11.87
miR-18a	0.0001	−14.58
miR-29b	0.0002	−15.81
miR-142-5p	0.0014	−17.92
miR-144	0.0202	−20.07
miR-423-3p	0.0024	−22.50
miR-142-3p	0.0003	−27.70
miR-19b	0.0001	−28.47
miR-19a	0.00003	−32.63
miR-32	0.0012	−35.70
miR-101	0.000001	−45.83
miR-141	0.00001	−136.81

**Table 2:** List of increased and decreased miRNA in slowly progressive IPF biopsies (n = 6) when compared to normal lung samples (n = 10) with p≤0.05.

miRNA	p-value	Fold increase/decrease
miR-155	0.02908	194.12
miR-128	0.01864	7.53
miR-125b	0.01713	5.33
miR-200c	0.02529	4.37
miR-181b	0.03575	−2.82
miR-210	0.01265	−3.31
miR-93	0.04296	−3.55
miR-376c	0.03582	−4.14
miR-126	0.03503	−4.77
let-7d	0.00081	−4.96
miR-29a	0.01268	−5.09
miR-186	0.01107	−5.14
miR-103	0.00474	−5.24
miR-424	0.02294	−5.56
miR-222	0.00053	−5.59
miR-223	0.03748	−5.92
miR-302c	0.04262	−6.82
miR-22	0.00365	−7.56
miR-20a	0.02531	−7.65
miR-17	0.01641	−7.98
miR-140-5p	0.01032	−8.68
miR-15a	0.00501	−8.81
miR-30e	0.01942	−9.08
miR-29b	0.00691	−10.02
miR-30b	0.00292	−10.55
miR-29c	0.00086	−11.30
miR-143	0.00061	−11.61
miR-106b	0.00049	−13.06
miR-18a	0.00081	−22.35
miR-142-3p	0.00172	−26.69
miR-142-5p	0.00220	−28.06
miR-19b	0.00131	−28.78
miR-130a	0.00001	−29.93
miR-101	0.00072	−34.27
miR-19a	0.00100	−35.17
miR-32	0.00358	−40.74
miR-144	0.01039	−81.52
miR-141	0.00003	−220.92

**Table 3:** List of increased and decreased miRNA in rapidly progressive IPF biopsies (n = 9) when compared with slow progressive IPF biopsies (n = 6) with p≤0.05.

miRNA	p-value	Fold increase/decrease
miR-302c	0.0057	10.56
miR-423-5p	0.0191	7.97
miR-210	0.0212	4.50
miR-376c	0.0264	3.84
miR-185	0.0262	2.94
miR-423-3p	0.0128	−97.68

### Dicer1 transcript expression did not differ between normal and IPF biopsies

**Figure 1:**
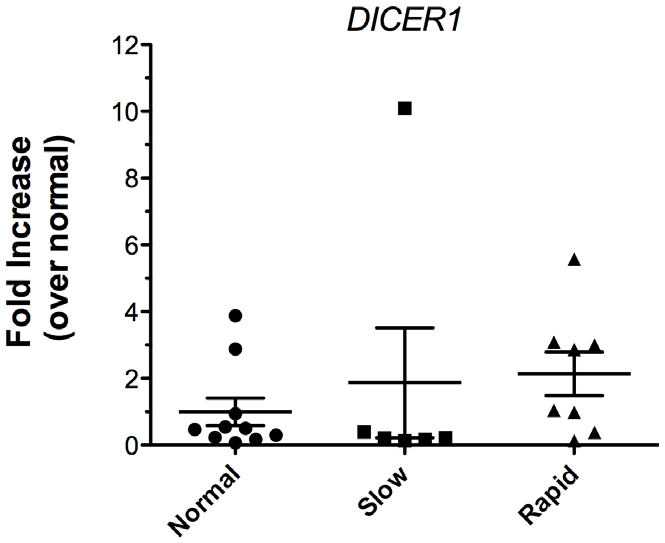
*DICER1* expression does not differ between normal and IPF biopsies. Total RNA from SLBs was analyzed by qRT-PCR for transcript expression. Relative expression values were normalized to the normal group (n = 10) and compared to the slowly progressive (n = 6) or rapidly progressive group (n = 9). Expression values were also normalized to GAPDH. Data are presented as mean ± SE.

Dicer1 is the ribonuclease required for the generation of mature miRNAs from pre-miRNAs [23]. Given that the majority of miRNA detected in both groups of IPF biopsies where significantly lower when compared to the normal biopsies, we speculated that altered *DICER1* expression might account for these findings. Total RNA from normal, slow IPF and rapid IPF biopsies was analyzed for *DICER1* expression using a qRT-PCR assay. *DICER1* transcript expression in the rapidly progressive IPF biopsies was only modestly increased over the average levels of this transcript in both slowly progressive IPF and normal biopsies (**Figure 1**). Thus, these findings did not support our hypothesis that alterations in *DICER1* expression might account for the differing miRNA generation in the IPF lung biopsies compared with normal lung biopsies.

### Numerous transcripts relevant to Epithelial-to-Mesenchymal Transition (EMT) were validated targets of the IPF miRNA profile

We next hypothesized that a decrease in miRNA expression might explain, in part, the increased expression of fibrosis-related mRNA transcripts observed in IPF biopsies compared with normal or non-fibrotic lung biopsies. Unfortunately, predicting miRNA gene targets is challenging because each target prediction method predicts thousands of mRNA transcripts per miRNA. Since each target prediction program's methodology differs, these programs also predict discordant mRNA transcripts for a given miRNA. To lessen the discordance in miRNA target prediction, we focused on compiling only experimentally validated miRNA targets after an analysis of interactions. A list of experimentally validated targets was compiled using the miRNA that differed between slowly progressive and rapidly progressive IPF biopsies compared to normal biopsies (**Supporting Table S1**). These data revealed that many transcripts relevant to fibrosis are validated targets of the miRNA present in the normal and IPF biopsy samples including many EMT-related genes (**Supporting Table S1**).

**Figure 2:**
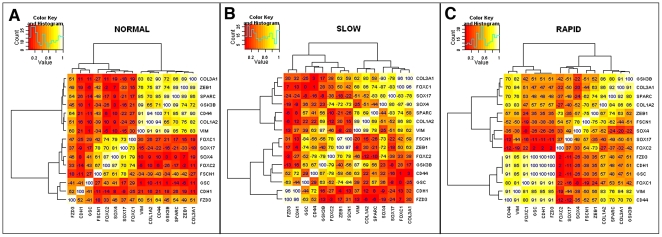
Correlations are shown between EMT-related genes in normal, slow IPF, and rapid IPF lung biopsies. The dCT values were clustered using the absolute value of correlation coefficients (distance measure) using hierarchical clustering to show the genes that are strongly related at each stage (NORMAL, SLOW and RAPID). Lighter colors represent greater correlation between genes. The larger the value indicated, the stronger the correlation. Negative values indicate a negative correlation.

To explore the relevance of EMT in IPF progression, a hierarchical clustering technique was used to determine the dynamics of gene correlation changes between normal and IPF, as well as between slow and rapid (**Figure 2**). Changes in the dynamics of gene correlation were more pronounced between the normal and rapid IPF groups and less pronounced between normal and slow IPF groups (**Figure 2**). Assessment of the clustering of specific EMT-related genes led to the observation that *GSK3b*, *COL3A1*, *SPARC* and *COL1A1* all correlated with *CD44* and *VIM* in normal lung biopsies, but not in rapid IPF lung biopsy samples or in slow IPF lung tissue (**Figure 2**). This suggested that the regulation of EMT-related genes was diminished or absent in IPF compared with normal biopsies.

**Figure 3:**
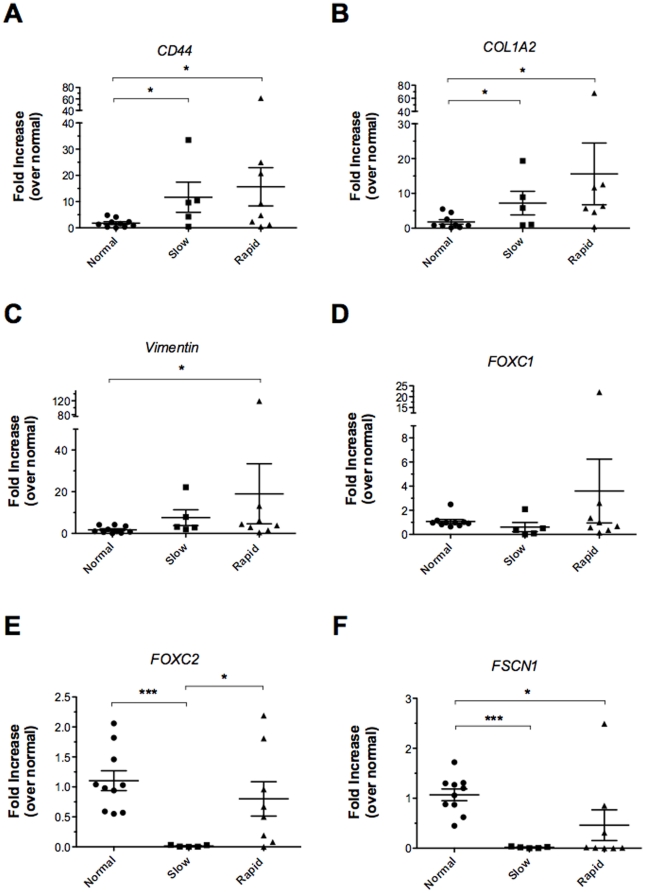
Differentially expressed transcripts of A) *CD44*, B) *COL1A2*, C) *VIM*, D) *FOXC1*, E) *FOXC2*, and F) *FSCN1* in rapidly progressive IPF and slowly progressive lung biopsies versus normal biopsies. Total RNA from SLBs was analyzed by qRT-PCR for transcript expression. Relative expression values were normalized to the normal group (n = 10) and compared to the slowly progressive (n = 6) or rapidly progressive group (n = 9). Expression values were also normalized to GAPDH. Data are presented as mean ± SE. Significant differences are shown as *P≤.05 and ***P≤.001.

We next determined whether EMT-related genes differed in the three groups of lung biopsies using TaqMan Array Microfluidic Cards, and these data are summarized in **Figure 3**. *CD44* and *COL1A2* mRNA levels were increased significantly in both slowly progressive and rapidly progressive IPF biopsies compared to normal biopsies (**Figure 3 A–B**). Messenger RNA levels of *vimentin* were significantly increased in rapidly progressive IPF biopsies compared with normal biopsies (**Figure 3C**). *FOXC1* transcripts did not differ among the three groups of biopsies (**Figure 3D**) while *FOXC2* expression was significantly higher in rapidly progressive IPF and normal biopsies compared with biopsies from slowly progressive IPF patients (**Figure 3E**). *FSCN1* (fascin homolog 1, actin bundling protein) mRNA levels were significantly decreased in slowly progressive biopsies and rapidly progressive biopsies in comparison to normal tissue (**Figure 3F**). Together, this quantitative PCR analysis suggested that although miRNA species targeting EMT-related mesenchymal markers were decreased in both groups of IPF patients, higher expression of certain of these EMT-associated markers (including *CD44, COL1A2, VIM, and FOXC1*) was apparent in rapid IPF versus slow IPF.

#### MicroRNAs in lung biopsy samples differentiate rapid from slow IPF

Further statistical analysis of miRNA expression in biopsies from both groups of IPF patients revealed that 5 miRNAs were increased in rapid IPF versus slow IPF, and one miRNA was decreased in rapid IPF versus slow IPF. A summary of these miRNA and their validated targets is shown in **Table 4**. Interestingly, few of these targets have been described in IPF aside from *HMGA2* [39], and none of the currently validated targets for these miRNA appear to be directly involved in EMT.

**Table 4:** List of experimentally validated miRNA gene targets using miRecords, which are increased or decreased in rapid IPF biopsies compared with slow IPF biopsies.

MiRNA	Symbol	Name
miR-302c	*ESR1*	estrogen receptor 1
miR-423-5p	none	
miR-210	*EFNA3*	Ephrin-A3
	*MNT*	MAX binding protein
	*CASP8AP2*	caspase-8-associated protein 2
miR-376c	none	
miR-185	*CDK6*	cyclin-dependent kinase 6
	***HMGA2***	**high mobility group AT-hook 2**
	*AKT1*	lv-akt murine thymoma viral oncogene homolog 1
	*CCNE1*	cyclin E1
	*CORO2B*	coronin, actin binding protein, 2B
miR-423-3p	none	

### Altered AGO1 and AGO2 transcript and AGO2 protein levels in rapid IPF biopsies

**Figure 4:**
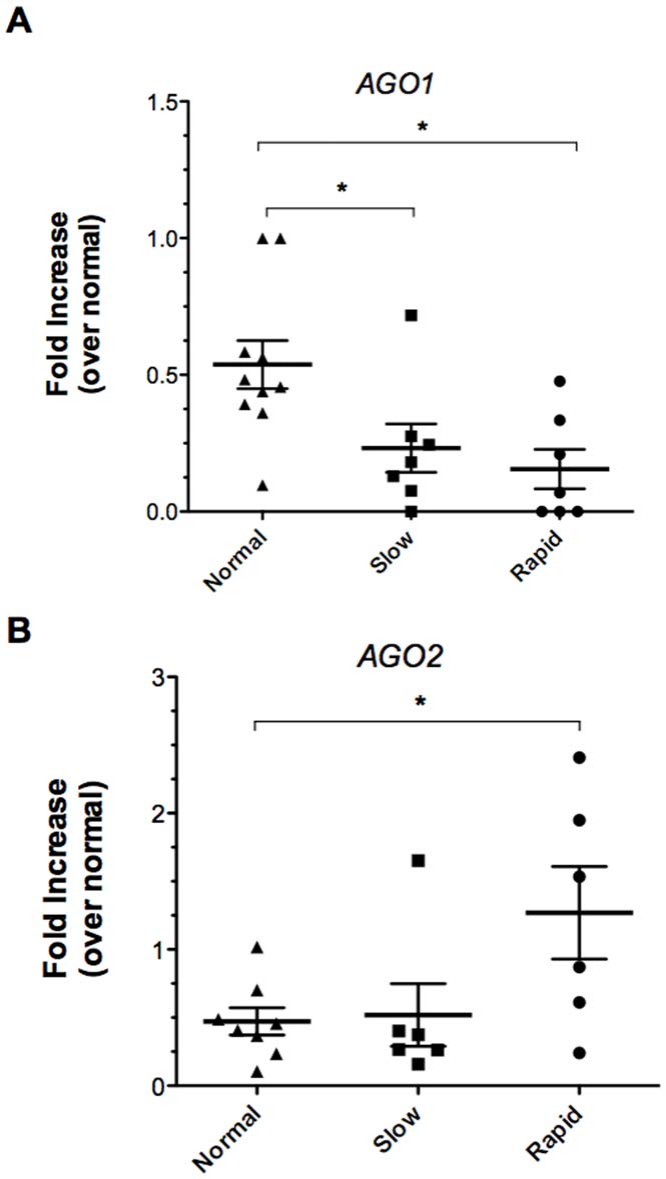
*Argonaute 1* (A) and Argonaute 2 (B) expression in normal and IPF patient lung biopsies. Total RNA from SLBs was analyzed by qRT-PCR for transcript expression. Relative expression values were normalized to the normal group (n = 10) and compared to the slowly progressive (n = 6) or rapidly progressive group (n = 9). Expression values were also normalized to GAPDH. Data are presented as mean ± SE. Significant differences are shown as *P≤0.05.

Analysis of the increased miRNA species in the IPF biopsies revealed that one of these miRNA (miR-128) targeted *AGO1*, which is a core component of the RNA induced silencing complex (RISC) [25], [26] (**Supporting Table S1**). Argonaute proteins function to bind the miRNA and correctly position it to recognize the appropriate mRNA targets [46]. Consequently, the expression levels of both *AGO1* and *AGO2* were analyzed at the transcript and protein level in IPF and normal biopsies. Compared to normal biopsies, *AGO1* transcript levels in slow and rapid IPF biopsies were significantly lower compared with levels in normal biopsies (**Figure 4A**). In contrast, AGO2 levels were significantly higher in rapid IPF biopsies compared with normal lung biopsies (**Figure 4B**). Together, these data suggested that *AGO1* and *AGO2* were differentially expressed in IPF compared with normal biopsies.

**Figure 5:**
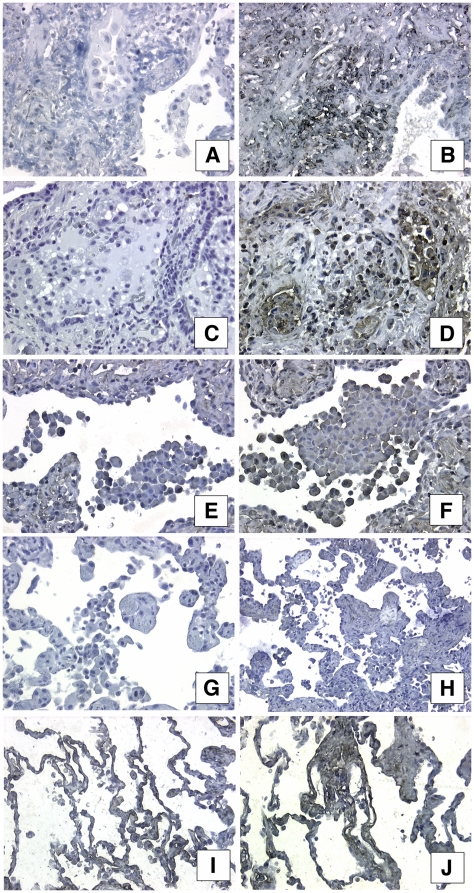
Immunohistochemical analysis of Argonaute2 (AGO2) in tissues sections from slowly progressive, rapidly progressive, or normal biopsies. Representative images of slowly progressive (**A–B, E–F**), rapidly progressive (**C–D, G–H**), and normal (**I–J**) biopsies stained with IgG control (**A, C, E, G, & I**) and anti-AGO2 antibody (**B, D, F, H, & J**) are shown. Images **A, B, C, & D** were stained with antibody concentrations of 20 µg/ml and images **E, F, G, H, I, & J** were stained with an antibody concentration of 4 µg/ml. Sections were counterstained with hematoxylin. Protein expression stains brown in this procedure (original magnification: ×200).

To explore these transcript obervations further at the protein level, immunohistochemical analysis was completed for AGO1 and AGO2 in surgical lung biopsy sections. Staining for *AGO1* revealed very little regardless of the antibody dilution or biopsy group, perhaps due to the lack of a suitable antibody for formalin-fixed tissues. However, AGO2 was detected in both slowly progressive and rapidly progressive sections when this antibody was used at a dilution of 20 µg/ml (**Figure 5 A–D**). However, upon lowering the anti-AGO2 antibody concentration (i.e. to 4 µg/ml), AGO2 protein expression was present in the same biopsies from both slowly progressive IPF and normal lung sections, but staining for this protein at this antibody dilution was absent in rapidly progressive biopsy sections (**Figure 5 E–J**). Semi-quantitative results from this immunohistochemical analysis are summarized in **Table 5**.

**Table 5:** Summary of semi-quantitative assessment of immunohistochemical staining for *Argonaute2* (antibody dilution of 4 µg/ml) in normal, slow, and rapid IPF biopsies.

Progression	Patient	Result of Staining
Normal	134	positive
Normal	142	positive
Slow	69	positive
Slow	76	positive
Slow	98	weak positive
Rapid	10	negative
Rapid	26	negative
Rapid	56	negative
Rapid	57	negative
Rapid	67	weak positive

### Decreased AGO1 transcript levels in rapid IPF biopsy-derived fibroblasts compared with normal biopsy-derived fibroblasts

**Figure 6:**
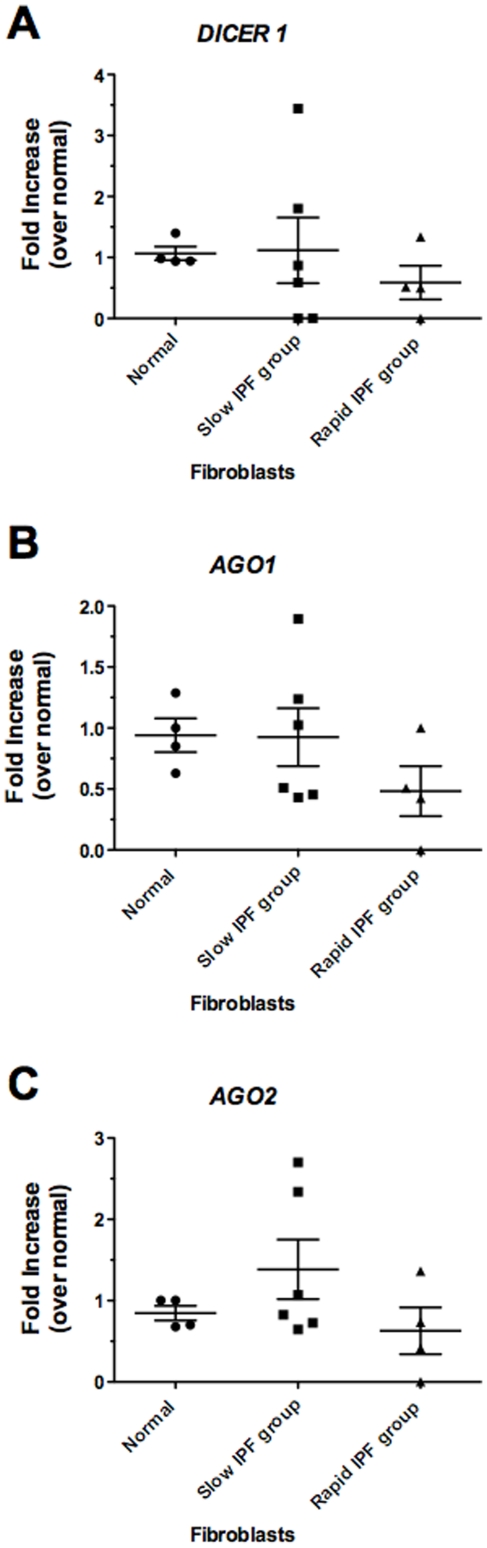
Transcript expression of A) *DICER1*, B) *AGO1*, & C) *AGO2* in normal, slow IPF, and rapid IPF patient fibroblasts. Fibroblasts cultured from normal, slow IPF, and rapid IPF lung biopsies were exposed to media alone. Total RNA from cells was analyzed by qRT-PCR for transcript expression 4 hours after treatment. Relative expression values were normalized to the normal group (n = 4) and compared to the slowly progressive (n = 6) or rapidly progressive group (n = 3). Expression values were also normalized to GAPDH. Data are presented as mean ± SE.

*DICER1*, *AGO1*, and *AGO2* expression were determined using quantitative PCR in cultured primary pulmonary fibroblasts grown from normal, slowly progressive IPF, or rapidly progressive IPF patient biopsies. These cells were treated with media alone and transcript levels were measured 4 h later. Median *DICER1* transcript levels were higher in the slowly progressive IPF fibroblast group compared with the other two groups (**Figure 6A**). *AGO1* expression was similar in the normal and slowly progressive groups but 50% reduction in the levels of this transcript were detected in cultures of fibroblasts from the rapidly progressive IPF group compared with the normal fibroblast lines (**Figure 6B**). Transcript levels of *AGO2* followed the same pattern of expression observed for *DICER1*; the highest levels of this transcript were detected in fibroblasts from the slowly progressive IPF group (**Figure 6C**). Overall, these data suggest that miRNA biosynthesis and RISC function might be altered in fibroblasts particularly from the rapidly progressive IPF group because of decreased expression of *DICER1* and *AGO1*, a catalytically active component of the machinery required for RISC function in fibroblasts from this group.

## Discussion

Microarray-based methodology has been previously used to identify transcript differences between biopsies from IPF and other fibrotic and/or non-fibrotic lung diseases [12], [13]. More recent attention has turned to genome-wide profiling of transcripts in biopsies taken from patients who experienced an acute exacerbation of IPF [45] or from those who exhibited varying progression of disease [14], [15]. While IPF progression has been defined in two published studies by Boon *et al* [14] and Selman *et al* [15], our definition of rapidly progressive IPF more closely matches that provided by Boon and colleagues [14]. Accordingly, rapidly progressive IPF was defined as those patients exhibiting a FVC and DL_CO_ decline of ≥10% and ≥15%, respectively, over the first 12 months after diagnosis. Our results demonstrate that quantitative differences in miRNA and mRNA expression in diagnostic surgical lung biopsies distinguish varying speeds of IPF progression. The lower expression levels of the majority of miRNA present in IPF biopsies compared with normal tissue closely mirrored findings in a wide variety of tumors compared with appropriate control tissues [47] [48]. Further, several EMT-related genes were found to be elevated in IPF biopsies (particularly those from rapidly progressive IPF patients) compared with normal biopsies. A comparison between slowly and rapidly progressive IPF biopsies revealed 5 miRNA that were significantly increased in rapid biopsies and 1 decreased when compared to slowly progressive biopsies. No differences in the expression of *Dicer1* among the biopsy or fibroblast lines were noted thus negating changes in miRNA processing as an explanation for the changes in miRNA levels between the groups. However, upon further investigation of miRNA processing components, we observed that *AGO1* levels in biopsies and fibroblast lines and AGO2 protein levels in biopsies were reduced in rapidly progressive IPF compared with normal samples. Together, these data suggest that IPF is characterized by the altered expression of miRNA and the decreased expression of key RISC components might explain the rapidly progressive form of this disease.

Our findings are consistent with a number of recently published studies directed at the characterization of miRNA expression and function in pulmonary fibrosis. First, a recent study by Pandit *et al* [39] examined miRNA levels in IPF, irrespective of progression, and observed that let-7d, miR-26, and members of the miR-30 family were all decreased in IPF biopsies compared with normal biopsies. They further demonstrated that TGF-β inhibited let-7d expression thereby driving the epithelial-mesenchymal transition (EMT; see below) and increased collagen deposition [39]. In the present study, we observed that members of the miR-30 and let-7d families were significantly decreased in both forms of IPF compared with normal biopsies. Second, Liu *et al* [40] reported that miR-21 was increased in the lungs of patients with IPF, irrespective again of disease progression, and that knocking down miR-21 attenuated fibrosis in a bleomycin-induced fibrotic model. They additionally found that an increase in miR-21 targets an inhibitory Smad, *Smad7*, causing an increase in TGF-β signaling and a fibrotic phenotype [40]. Interestingly, our results revealed that miR-21 was significantly increased in rapidly progressive IPF biopsies compared with normal biopsies. Third, Cushing *et al* [41] used a bleomycin-induced fibrotic mouse model and found that the expression of miR-29 family miRNA was reduced in fibrotic lungs, which corresponded with an increase in collagens and ECM-related genes like laminins and integrins. In our study, we observed significant decreases in miR-29b and miR-29c in slowly progressive and rapidly progressive IPF patients compared to normal. Also concordant with this previous study, we detected an increase in *COL1A2* (a miR-29b and miR-29c target) in IPF patients. Thus, the findings from the present study coincide with other published findings regarding the differential expression of miRNA in clinical and experimental fibrosis.

*DICER1* enzymatic activity is necessary for miRNA biogenesis and loss of *DICER1* expression through gene silencing and knockout approaches has been shown to cause aberrant or decreased endothelial cell proliferation [49], hair follicle development [50], T cell development [51], lung epithelium morphogenesis [52], and reproductive development [53]. Loss of function mutations in *DICER1* is also associated with familial pleuropulmonary blastoma [54], and its reduced expression contributes to lung tumorigenesis [55], [56]. In the majority of these disorders, the reduction of *DICER1* caused overall reductions in mature miRNA levels, which contributes to the loss of transcript regulation. However, analysis of *DICER1* expression revealed no differences in the amounts of this transcript amongst the biopsies and fibroblast lines analyzed, leading us to presently conclude that changes in the expression of *DICER1* do not appear to explain the differential miRNA levels in IPF versus normal.

EMT is the transition of differentiated epithelial cells into motile mesenchymal cells, and this process is prominent in both experimental and clinical pulmonary fibrosis [57]–[59]. Previous studies have shown that miRNA regulation is critical in EMT. For example, members of the miR-200 family (miR-200a, miR-200b, miR-200c, miR-141, and miR-429) and miR-205 are decreased when cells undergo EMT [60]. This group of miRNAs target *ZEB1* and *SIP1*, which repress E-cadherin and promote mesenchymal marker expression. As expected [14], [15], many of the elevated mRNA transcripts detected in the IPF biopsies we analyzed herein have been implicated in EMT. Further, transcripts such as *CD44*, *COL1A2*, *VIM*, and *FOXC2* were significantly increased in rapidly progressive IPF over normal and/or slowly progressive IPF biopsies. *CD44* is a membrane associated cellular adhesion receptor that plays a role in remodeling the lung [61] through its ability to co-localize and bind with other EMT-related proteins [62]. *COL1A2* has been previously described in IPF [45], and this gene was significantly increased in both forms of IPF, perhaps due to the decreased miR-29b and miR-29c in these biopsies. *Vimentin* is a definitive marker for the meshchymal cells derived from epithelium [63] and an experimentally validated target of miR-17. However, miR-17 was significantly decreased in both forms of IPF compared with normal biopsies while levels of *VIM* were significantly increased only in rapidly progressive IPF. These data suggested that levels of a particular miRNA might be less important than the processing efficiency of a particular miRNA. Together, these data highlight that transcripts associated with EMT were significantly elevated in IPF, particularly the rapidly progressive form of this disease. The impact of differential miRNA expression on EMT during IPF progression is presently not clear and requires further investigation.

Argonaute proteins bind miRNA and position it in a conformation that promotes mRNA target recognition [46]. They are essential in every functional RISC and *AGO2* is the catalytically active “slicer” that cleaves mRNA transcripts [26]. Expanding on the observation that differential miRNA expression did not fully explain the regulation of EMT-related genes in IPF of variable progression, we investigated the expression of AGO1 and AGO2 in biopsies and fibroblasts derived from the same biopsies. The present study suggested that there was both a defect in transcript and protein expression of these RISC components both in biopsies and in cultured primary fibroblasts. Thus, the impairment of miRNA-mediated gene silencing in rapidly progressive forms of IPF could perhaps contribute to disease progression. Future studies will be directed at determining what mechanisms regulate Argonaute expression in IPF and whether RISC function can be restored during fibrosis.

In summary, our results demonstrate that miRNA profiling in diagnostic surgical lung biopsies differentiates normal from IPF, and rapidly progressive IPF from slowly progressive IPF. Further analysis of miRNA levels in circulating cells as a means of ascertaining IPF disease progression is certainly warranted given the present findings. Alterations in circulating miRNAs have been detected in other diseases such as cancer. Finally, our results indicate that aberrations in the miRNA processing pathway might be the cause of altered transcript expression leading to the fibrotic phenotype in IPF and the differing speed of progression of this disease.

## Supporting Information

Table S1 This table is a list of the experimentally validated gene targets compiled using the miRNA species that differed between slowly progressive and rapidly progressive IPF biopsies compared with normal lung biopsies.
(DOCX)
