# Supplementary material for: A Micro RNA Processing Defect in Rapidly Progressing Idiopathic Pulmonary Fibrosis
Source: PLoS One. 2011 Jun 21;6(6):e21253. doi: 10.1371/journal.pone.0021253 (PMC3119674; doi:10.1371/journal.pone.0021253)
Supplement: Table S1 — This table is a list of the experimentally validated gene targets compiled using the miRNA species that differed between slowly progressive and rapidly progressive IPF biopsies compared with normal lung biopsies. (DOCX) [file pone.0021253.s001.docx]

**Supporting Table S1:** List of experimentally validated miRNA Gene Targets using miRecords that are increased/decreased in slow IPF biopsies compared with normal lung samples, and rapid IPF biopsies compared with normal lung samples.

|  |  |  |  |  |  |  |  |  |  |  |
| --- | --- | --- | --- | --- | --- | --- | --- | --- | --- | --- |
|  | **Validated targets that differ in slow IPF vs. normal lung biopsies** | |  |  |  |  |  |  |  |  |
|  |  |  |  |  |  |  |  |  |  |  |
|  | **Symbol** | **Name** |  |  |  |  |  |  |  |  |
| miR-155 | AGTR1 | Angiotensin II receptor, type 1 |  |  |  |  |  |  |  |  |
|  | BACH1 | BTB and CNC homology 1, basic leucine zipper transcription factor 1 |  |  |  |  |  |  |  |  |
|  | LDOC1 | Leucine zipper, down-regulated in cancer 1 |  |  |  |  |  |  |  |  |
|  | MATR3 | Matrin 3 |  |  |  |  |  |  |  |  |
|  | TM6SF1 | Transmembrane 6 superfamily member 1 |  |  |  |  |  |  |  |  |
|  | AGTR1 | Angiotensin II receptor, type 1 |  |  |  |  |  |  |  |  |
|  | RHOA | Ras homolog gene family, member A |  |  |  |  |  |  |  |  |
|  | ETS1 | V-ets erythroblastosis virus E26 oncogene homolog 1 (avian) |  |  |  |  |  |  |  |  |
|  | MEIS1 | Meis homeobox 1 |  |  |  |  |  |  |  |  |
|  | MAP3K7IP2 | Mitogen-activated protein kinase kinase kinase 7 interacting protein 2 |  |  |  |  |  |  |  |  |
|  | CYR61 | Cysteine-rich, angiogenic inducer, 61 |  |  |  |  |  |  |  |  |
|  | MyD88 | Myeloid differentiation primary response gene (88) |  |  |  |  |  |  |  |  |
|  | FOXO3 | Forkhead box O3 |  |  |  |  |  |  |  |  |
|  | SOCS1 | Suppressor of cytokine signaling 1 |  |  |  |  |  |  |  |  |
|  | CCND1 | Cyclin D1 |  |  |  |  |  |  |  |  |
|  | IKBKE | Inhibitor of kappa light polypeptide gene enhancer in B-cells, kinase epsilon |  |  |  |  |  |  |  |  |
|  | KGF |  |  |  |  |  |  |  |  |  |
|  | SPI1 | Spleen focus forming virus (SFFV) proviral integration oncogene spi1 |  |  |  |  |  |  |  |  |
|  | FADD | Fas (TNFRSF6)-associated via death domain |  |  |  |  |  |  |  |  |
|  | JARID2 | Jumonji, AT rich interactive domain 2 |  |  |  |  |  |  |  |  |
|  | MeCP2 | Methyl CpG binding protein 2 (Rett syndrome) |  |  |  |  |  |  |  |  |
|  | CEBPB | CCAAT/enhancer binding protein (C/EBP), beta |  |  |  |  |  |  |  |  |
|  | SHIP |  |  |  |  |  |  |  |  |  |
|  | SMAD2 | SMAD family member 2 |  |  |  |  |  |  |  |  |
|  |  |  |  |  |  |  |  |  |  |  |
| miR-128 | E2F3 | E2F transcription factor 3 |  |  |  |  |  |  |  |  |
|  | NTRK3 | Neurotrophic tyrosine kinase, receptor, type 3 |  |  |  |  |  |  |  |  |
|  | BMI1 | BMI1 polycomb ring finger oncogene |  |  |  |  |  |  |  |  |
|  | ADORA2B | Adenosine A2b receptor |  |  |  |  |  |  |  |  |
|  | DBI | Diazepam binding inhibitor (GABA receptor modulator, acyl-Coenzyme A binding protein) |  |  |  |  |  |  |  |  |
|  | AGO1 | Argonaute 1 |  |  |  |  |  |  |  |  |
|  | LDLR | Low density lipoprotein receptor (familial hypercholesterolemia) |  |  |  |  |  |  |  |  |
|  | DCX | Doublecortex; lissencephaly, X-linked (doublecortin) |  |  |  |  |  |  |  |  |
|  | RELN | Reelin |  |  |  |  |  |  |  |  |
|  | MLL | Myeloid/lymphoid or mixed-lineage leukemia (trithorax homolog, Drosophila) |  |  |  |  |  |  |  |  |
|  | AF4 |  |  |  |  |  |  |  |  |  |
|  | TXNIP | Thioredoxin interacting protein |  |  |  |  |  |  |  |  |
|  |  |  |  |  |  |  |  |  |  |  |
| miR-125b | SMO | Smoothened homolog (Drosophila) |  |  |  |  |  |  |  |  |
|  | BMF | Bcl2 modifying factor |  |  |  |  |  |  |  |  |
|  | BMPR1B | Bone morphogenetic protein receptor, type IB |  |  |  |  |  |  |  |  |
|  | HuR |  |  |  |  |  |  |  |  |  |
|  | CDK6 | Cyclin-dependent kinase 6 |  |  |  |  |  |  |  |  |
|  | CDC25A | Cell division cycle 25 homolog A (S. pombe) |  |  |  |  |  |  |  |  |
|  | KRT |  |  |  |  |  |  |  |  |  |
|  | ST18 | Suppression of tumorigenicity 18 (breast carcinoma) (zinc finger protein) |  |  |  |  |  |  |  |  |
|  | DICER1 | Dicer1, Dcr-1 homolog (Drosophila) |  |  |  |  |  |  |  |  |
|  | CDKN2A | Cyclin-dependent kinase inhibitor 2A (melanoma, p16, inhibits CDK4) |  |  |  |  |  |  |  |  |
|  |  |  |  |  |  |  |  |  |  |  |
| miR-200c | ZEB1 | Zinc finger E-box binding homeobox 1 |  |  |  |  |  |  |  |  |
|  | ERRFI1 | ERBB receptor feedback inhibitor 1 |  |  |  |  |  |  |  |  |
|  |  |  |  |  |  |  |  |  |  |  |
| miR-181b | pcaf | P300/CBP-associated factor |  |  |  |  |  |  |  |  |
|  | Tcl1 |  |  |  |  |  |  |  |  |  |
|  | VSNL1 | Visinin-like 1 |  |  |  |  |  |  |  |  |
|  | GRIA2 | Glutamate receptor, ionotropic, AMPA 2 |  |  |  |  |  |  |  |  |
|  | CDX2 | Caudal type homeobox 2 |  |  |  |  |  |  |  |  |
|  | GATA6 | GATA binding protein 6 |  |  |  |  |  |  |  |  |
|  | NLK | Nemo-like kinase |  |  |  |  |  |  |  |  |
|  | ESR1 | Estrogen receptor 1 |  |  |  |  |  |  |  |  |
|  | PLAG1 | Pleiomorphic adenoma gene 1 |  |  |  |  |  |  |  |  |
|  |  |  |  |  |  |  |  |  |  |  |
| miR-210 | Ephrin-A3 |  |  |  |  |  |  |  |  |  |
|  | MNT | MAX binding protein |  |  |  |  |  |  |  |  |
|  | CASP8AP2 | CASP8 associated protein 2 |  |  |  |  |  |  |  |  |
|  |  |  |  |  |  |  |  |  |  |  |
| miR-93 | p21 |  |  |  |  |  |  |  |  |  |
|  | E2F1 | E2F transcription factor 1 |  |  |  |  |  |  |  |  |
|  |  |  |  |  |  |  |  |  |  |  |
| miR-376c | none |  |  |  |  |  |  |  |  |  |
|  |  |  |  |  |  |  |  |  |  |  |
| miR-126 | VCAM-1 |  |  |  |  |  |  |  |  |  |
|  | Crk II |  |  |  |  |  |  |  |  |  |
|  | p85beta |  |  |  |  |  |  |  |  |  |
|  | IRS-1 |  |  |  |  |  |  |  |  |  |
|  | VEGF-A |  |  |  |  |  |  |  |  |  |
|  | TOM1 | Target of myb1 (chicken) |  |  |  |  |  |  |  |  |
|  | prostein |  |  |  |  |  |  |  |  |  |
|  |  |  |  |  |  |  |  |  |  |  |
| let-7d | DICER1 | Dicer1, Dcr-1 homolog (Drosophila) |  |  |  |  |  |  |  |  |
|  | DAD3R |  |  |  |  |  |  |  |  |  |
|  | DAD1 | Defender against cell death 1 |  |  |  |  |  |  |  |  |
|  | EIF4G2 | Eukaryotic translation initiation factor 4 gamma, 2 |  |  |  |  |  |  |  |  |
|  | RABGAP1L | RAB GTPase activating protein 1-like |  |  |  |  |  |  |  |  |
|  | SMOX | Spermine oxidase |  |  |  |  |  |  |  |  |
|  |  |  |  |  |  |  |  |  |  |  |
| miR-29a | DNMT3B | DNA (cytosine-5-)-methyltransferase 3 beta |  |  |  |  |  |  |  |  |
|  | SPARC | Secreted protein, acidic, cysteine-rich (osteonectin) |  |  |  |  |  |  |  |  |
|  | BACE1 | Beta-site APP-cleaving enzyme 1 |  |  |  |  |  |  |  |  |
|  | PIK3R1 | Phosphoinositide-3-kinase, regulatory subunit 1 (p85 alpha) |  |  |  |  |  |  |  |  |
|  | CDC42 | Cell division cycle 42 (GTP binding protein, 25kDa) |  |  |  |  |  |  |  |  |
|  | B7-H3 |  |  |  |  |  |  |  |  |  |
|  | MCL-1 |  |  |  |  |  |  |  |  |  |
|  | CXXC6 | CXXC finger 6 |  |  |  |  |  |  |  |  |
|  | CDK6 | Cyclin-dependent kinase 6 |  |  |  |  |  |  |  |  |
|  | COL3A1 | Collagen, type III, alpha 1 (Ehlers-Danlos syndrome type IV, autosomal dominant) |  |  |  |  |  |  |  |  |
|  |  |  |  |  |  |  |  |  |  |  |
| miR-186 | FOXO1 | Forkhead box O1 |  |  |  |  |  |  |  |  |
|  |  |  |  |  |  |  |  |  |  |  |
| miR-103 | FBXW1B |  |  |  |  |  |  |  |  |  |
|  | serbp1 | SERPINE1 mRNA binding protein 1 |  |  |  |  |  |  |  |  |
|  | ICOS | Inducible T-cell co-stimulator |  |  |  |  |  |  |  |  |
|  |  |  |  |  |  |  |  |  |  |  |
| miR-424 | NFI-A |  |  |  |  |  |  |  |  |  |
|  | PLAG1 | Pleiomorphic adenoma gene 1 |  |  |  |  |  |  |  |  |
|  | FGFR1 | Fibroblast growth factor receptor 1 (fms-related tyrosine kinase 2, Pfeiffer syndrome) |  |  |  |  |  |  |  |  |
|  | MEK1 |  |  |  |  |  |  |  |  |  |
|  | Galectin-3 |  |  |  |  |  |  |  |  |  |
|  |  |  |  |  |  |  |  |  |  |  |
| miR-222 | KIT | V-kit Hardy-Zuckerman 4 feline sarcoma viral oncogene homolog |  |  |  |  |  |  |  |  |
|  | p27Kip1 |  |  |  |  |  |  |  |  |  |
|  | p27 |  |  |  |  |  |  |  |  |  |
|  | p27Kip1 |  |  |  |  |  |  |  |  |  |
|  | ER alpha |  |  |  |  |  |  |  |  |  |
|  | BIM |  |  |  |  |  |  |  |  |  |
|  | MMP1 | Matrix metallopeptidase 1 (interstitial collagenase) |  |  |  |  |  |  |  |  |
|  | SOD2 | Superoxide dismutase 2, mitochondrial |  |  |  |  |  |  |  |  |
|  | CDKN1C | Cyclin-dependent kinase inhibitor 1C (p57, Kip2) |  |  |  |  |  |  |  |  |
|  | PPP2R2A | Protein phosphatase 2 (formerly 2A), regulatory subunit B, alpha isoform |  |  |  |  |  |  |  |  |
|  | c-fos |  |  |  |  |  |  |  |  |  |
|  |  |  |  |  |  |  |  |  |  |  |
| miR-223 | NFI-A |  |  |  |  |  |  |  |  |  |
|  | STMN1 | Stathmin 1/oncoprotein 18 |  |  |  |  |  |  |  |  |
|  | LMO2 | LIM domain only 2 (rhombotin-like 1) |  |  |  |  |  |  |  |  |
|  | IRS-1 |  |  |  |  |  |  |  |  |  |
|  | RhoB | Ras homolog gene family, member B |  |  |  |  |  |  |  |  |
|  |  |  |  |  |  |  |  |  |  |  |
| miR-302c | ESR1 | Estrogen receptor 1 |  |  |  |  |  |  |  |  |
|  |  |  |  |  |  |  |  |  |  |  |
| miR-22 | PTEN | Phosphatase and tensin homolog (mutated in multiple advanced cancers 1) |  |  |  |  |  |  |  |  |
|  | ESR1 | Estrogen receptor 1 |  |  |  |  |  |  |  |  |
|  | PPARA | Peroxisome proliferator-activated receptor alpha |  |  |  |  |  |  |  |  |
|  | BMP7 | Bone morphogenetic protein 7 (osteogenic protein 1) |  |  |  |  |  |  |  |  |
|  | MAX | MYC associated factor X |  |  |  |  |  |  |  |  |
|  |  |  |  |  |  |  |  |  |  |  |
| miR-20a | E2F1 | E2F transcription factor 1 |  |  |  |  |  |  |  |  |
|  | AML1 |  |  |  |  |  |  |  |  |  |
|  | cyclin D1 |  |  |  |  |  |  |  |  |  |
|  | BMPRII |  |  |  |  |  |  |  |  |  |
|  | BCL2 | B-cell CLL/lymphoma 2 |  |  |  |  |  |  |  |  |
|  | MEF2D | Myocyte enhancer factor 2D |  |  |  |  |  |  |  |  |
|  | MAP3K12 | Mitogen-activated protein kinase kinase kinase 12 |  |  |  |  |  |  |  |  |
|  | JAK1 | Janus kinase 1 (a protein tyrosine kinase) |  |  |  |  |  |  |  |  |
|  | BIM |  |  |  |  |  |  |  |  |  |
|  | IL-8 |  |  |  |  |  |  |  |  |  |
|  |  |  |  |  |  |  |  |  |  |  |
| miR-17 | AIB1 |  |  |  |  |  |  |  |  |  |
|  | AML1 |  |  |  |  |  |  |  |  |  |
|  | VEGF |  |  |  |  |  |  |  |  |  |
|  | cyclin D1 |  |  |  |  |  |  |  |  |  |
|  | BMPRII |  |  |  |  |  |  |  |  |  |
|  | BCL2 | B-cell CLL/lymphoma 2 |  |  |  |  |  |  |  |  |
|  | MEF2D | Myocyte enhancer factor 2D |  |  |  |  |  |  |  |  |
|  | MAP3K12 | Mitogen-activated protein kinase kinase kinase 12 |  |  |  |  |  |  |  |  |
|  | Vim | Vimentin |  |  |  |  |  |  |  |  |
|  | CCND1 | Cyclin D1 |  |  |  |  |  |  |  |  |
|  | JAK1 | Janus kinase 1 (a protein tyrosine kinase) |  |  |  |  |  |  |  |  |
|  | CDKN1A | Cyclin-dependent kinase inhibitor 1A (p21, Cip1) |  |  |  |  |  |  |  |  |
|  | EDG1 | Endothelial differentiation, sphingolipid G-protein-coupled receptor, 1 |  |  |  |  |  |  |  |  |
|  | BIM |  |  |  |  |  |  |  |  |  |
|  | IL-8 |  |  |  |  |  |  |  |  |  |
|  |  |  |  |  |  |  |  |  |  |  |
| miR-140-5p | VEGF |  |  |  |  |  |  |  |  |  |
|  | HDAC4 | Histone deacetylase 4 |  |  |  |  |  |  |  |  |
|  | IGFBP-5 |  |  |  |  |  |  |  |  |  |
|  |  |  |  |  |  |  |  |  |  |  |
| miR-15a | DMTF1 | Cyclin D binding myb-like transcription factor 1 |  |  |  |  |  |  |  |  |
|  | BCL2 | B-cell CLL/lymphoma 2 |  |  |  |  |  |  |  |  |
|  | VEGF |  |  |  |  |  |  |  |  |  |
|  | PDCD4 | Programmed cell death 4 (neoplastic transformation inhibitor) |  |  |  |  |  |  |  |  |
|  | RAB21 | RAB21, member RAS oncogene family |  |  |  |  |  |  |  |  |
|  | IGSF4 |  |  |  |  |  |  |  |  |  |
|  | SCAP2 |  |  |  |  |  |  |  |  |  |
|  | Rab9B | RAB9B, member RAS oncogene family |  |  |  |  |  |  |  |  |
|  | Tpi1 | Triosephosphate isomerase 1 |  |  |  |  |  |  |  |  |
|  | c-Myb |  |  |  |  |  |  |  |  |  |
|  | CCND1 | Cyclin D1 |  |  |  |  |  |  |  |  |
|  | BMI-1 |  |  |  |  |  |  |  |  |  |
|  |  |  |  |  |  |  |  |  |  |  |
| miR-30e | UBE2I | Ubiquitin-conjugating enzyme E2I (UBC9 homolog, yeast) |  |  |  |  |  |  |  |  |
|  |  |  |  |  |  |  |  |  |  |  |
| miR-29b | Tcl1 |  |  |  |  |  |  |  |  |  |
|  | MCL1 | Myeloid cell leukemia sequence 1 (BCL2-related) |  |  |  |  |  |  |  |  |
|  | DNMT3A | DNA (cytosine-5-)-methyltransferase 3 alpha |  |  |  |  |  |  |  |  |
|  | DNMT3B | DNA (cytosine-5-)-methyltransferase 3 beta |  |  |  |  |  |  |  |  |
|  | BACE1 | Beta-site APP-cleaving enzyme 1 |  |  |  |  |  |  |  |  |
|  | CDC42 | Cell division cycle 42 (GTP binding protein, 25kDa) |  |  |  |  |  |  |  |  |
|  | PIK3R1 | Phosphoinositide-3-kinase, regulatory subunit 1 (p85 alpha) |  |  |  |  |  |  |  |  |
|  | SP1 | Sp1 transcription factor |  |  |  |  |  |  |  |  |
|  | MCL-1 |  |  |  |  |  |  |  |  |  |
|  | CXXC6 | CXXC finger 6 |  |  |  |  |  |  |  |  |
|  | CDK6 | Cyclin-dependent kinase 6 |  |  |  |  |  |  |  |  |
|  | COL1A1 | Collagen, type I, alpha 1 |  |  |  |  |  |  |  |  |
|  | SP1 | Sp1 transcription factor |  |  |  |  |  |  |  |  |
|  | COL3A1 | Collagen, type III, alpha 1 (Ehlers-Danlos syndrome type IV, autosomal dominant) |  |  |  |  |  |  |  |  |
|  |  |  |  |  |  |  |  |  |  |  |
| miR-30b | none |  |  |  |  |  |  |  |  |  |
|  |  |  |  |  |  |  |  |  |  |  |
| miR-29c | DNMT3B | DNA (cytosine-5-)-methyltransferase 3 beta |  |  |  |  |  |  |  |  |
|  | COL3A1 | Collagen, type III, alpha 1 (Ehlers-Danlos syndrome type IV, autosomal dominant) |  |  |  |  |  |  |  |  |
|  | COL4A1 | Collagen, type IV, alpha 1 |  |  |  |  |  |  |  |  |
|  | COL15A1 | Collagen, type XV, alpha 1 |  |  |  |  |  |  |  |  |
|  | LAMC1 | Laminin, gamma 1 (formerly LAMB2) |  |  |  |  |  |  |  |  |
|  | TDG | Thymine-DNA glycosylase |  |  |  |  |  |  |  |  |
|  | FUSIP1 | FUS interacting protein (serine/arginine-rich) 1 |  |  |  |  |  |  |  |  |
|  | COL1A1 | Collagen, type I, alpha 1 |  |  |  |  |  |  |  |  |
|  | COL1A2 | Collagen, type I, alpha 2 |  |  |  |  |  |  |  |  |
|  | COL4A2 | Collagen, type IV, alpha 2 |  |  |  |  |  |  |  |  |
|  | fibrillin 1 |  |  |  |  |  |  |  |  |  |
|  | CDC42 | Cell division cycle 42 (GTP binding protein, 25kDa) |  |  |  |  |  |  |  |  |
|  | PIK3R1 | Phosphoinositide-3-kinase, regulatory subunit 1 (p85 alpha) |  |  |  |  |  |  |  |  |
|  |  |  |  |  |  |  |  |  |  |  |
| miR-143 | ERK5 |  |  |  |  |  |  |  |  |  |
|  | KRAS | V-Ki-ras2 Kirsten rat sarcoma viral oncogene homolog |  |  |  |  |  |  |  |  |
|  | ERK5 |  |  |  |  |  |  |  |  |  |
|  | FNDC3b | Fibronectin type III domain containing 3B |  |  |  |  |  |  |  |  |
|  | DNMT3A | DNA (cytosine-5-)-methyltransferase 3 alpha |  |  |  |  |  |  |  |  |
|  |  |  |  |  |  |  |  |  |  |  |
| miR-106b | ITCH | Itchy homolog E3 ubiquitin protein ligase (mouse) |  |  |  |  |  |  |  |  |
|  | CDKN1A | Cyclin-dependent kinase inhibitor 1A (p21, Cip1) |  |  |  |  |  |  |  |  |
|  | E2F1 | E2F transcription factor 1 |  |  |  |  |  |  |  |  |
|  |  |  |  |  |  |  |  |  |  |  |
| miR-18a | ESR1 | Estrogen receptor 1 |  |  |  |  |  |  |  |  |
|  | thbs1 | Thrombospondin 1 |  |  |  |  |  |  |  |  |
|  | BIM |  |  |  |  |  |  |  |  |  |
|  | CTGF | Connective tissue growth factor |  |  |  |  |  |  |  |  |
|  |  |  |  |  |  |  |  |  |  |  |
| miR-142-3p | none |  |  |  |  |  |  |  |  |  |
|  |  |  |  |  |  |  |  |  |  |  |
| miR-142-5p | none |  |  |  |  |  |  |  |  |  |
|  |  |  |  |  |  |  |  |  |  |  |
| miR-19b | ESR1 | Estrogen receptor 1 |  |  |  |  |  |  |  |  |
|  | CTGF | Connective tissue growth factor |  |  |  |  |  |  |  |  |
|  |  |  |  |  |  |  |  |  |  |  |
| miR-130a | MCSF |  |  |  |  |  |  |  |  |  |
|  | MAFB | V-maf musculoaponeurotic fibrosarcoma oncogene homolog B (avian) |  |  |  |  |  |  |  |  |
|  | GAX |  |  |  |  |  |  |  |  |  |
|  | HOXA5 | Homeobox A5 |  |  |  |  |  |  |  |  |
|  |  |  |  |  |  |  |  |  |  |  |
| miR-101 | N-MYC |  |  |  |  |  |  |  |  |  |
|  | ICOS | Inducible T-cell co-stimulator |  |  |  |  |  |  |  |  |
|  | EZH2 | Enhancer of zeste homolog 2 (Drosophila) |  |  |  |  |  |  |  |  |
|  | Cox-2 |  |  |  |  |  |  |  |  |  |
|  | MCL1 | Myeloid cell leukemia sequence 1 (BCL2-related) |  |  |  |  |  |  |  |  |
|  |  |  |  |  |  |  |  |  |  |  |
| miR-19a | PTEN | Phosphatase and tensin homolog (mutated in multiple advanced cancers 1) |  |  |  |  |  |  |  |  |
|  | BMPRII |  |  |  |  |  |  |  |  |  |
|  | NR4A2 | Nuclear receptor subfamily 4, group A, member 2 |  |  |  |  |  |  |  |  |
|  | ERBB4 | V-erb-a erythroblastic leukemia viral oncogene homolog 4 (avian) |  |  |  |  |  |  |  |  |
|  | ESR1 | Estrogen receptor 1 |  |  |  |  |  |  |  |  |
|  | CCND1 | Cyclin D1 |  |  |  |  |  |  |  |  |
|  | thbs1 | Thrombospondin 1 |  |  |  |  |  |  |  |  |
|  | BIM |  |  |  |  |  |  |  |  |  |
|  | CTGF | Connective tissue growth factor |  |  |  |  |  |  |  |  |
|  |  |  |  |  |  |  |  |  |  |  |
| miR-32 | none |  |  |  |  |  |  |  |  |  |
|  |  |  |  |  |  |  |  |  |  |  |
| miR-144 | none |  |  |  |  |  |  |  |  |  |
|  |  |  |  |  |  |  |  |  |  |  |
| miR-141 | Clock | Clock homolog (mouse) |  |  |  |  |  |  |  |  |
|  | TGF beta2 |  |  |  |  |  |  |  |  |  |
|  | SIP1 | Survival of motor neuron protein interacting protein 1 |  |  |  |  |  |  |  |  |
|  | MKK4 |  |  |  |  |  |  |  |  |  |
|  | ERBB2IP | Erbb2 interacting protein |  |  |  |  |  |  |  |  |
|  | BAP1 | BRCA1 associated protein-1 (ubiquitin carboxy-terminal hydrolase) |  |  |  |  |  |  |  |  |
|  | KLHL20 | Kelch-like 20 (Drosophila) |  |  |  |  |  |  |  |  |
|  | PTPRD | Protein tyrosine phosphatase, receptor type, D |  |  |  |  |  |  |  |  |
|  | ELMO2 | Engulfment and cell motility 2 |  |  |  |  |  |  |  |  |
|  | WDR37 | WD repeat domain 37 |  |  |  |  |  |  |  |  |
|  | | | |  |  |  |  |  | ER alpha |  |
|  | | | |  |  |  |  |  | BIM |  |
|  | | | |  |  |  |  |  | MMP1 | Matrix metallopeptidase 1 (interstitial collagenase) |
|  | | | |  |  |  |  |  | SOD2 | Superoxide dismutase 2, mitochondrial |
|  | | | |  |  |  |  |  | CDKN1C | Cyclin-dependent kinase inhibitor 1C (p57, Kip2) |

| \| **Validated targets that differ in rapid IPF biopsies vs. normal lung biopsies** \| \| \| \| --- \| --- \| --- \| \|  \|  \|  \| \|  \| **Symbol** \| **Name** \| \| miR-423-5p \| none \|  \| \|  \|  \|  \| \| miR-155 \| AGTR1 \| Angiotensin II receptor, type 1 \| \|  \| BACH1 \| BTB and CNC homology 1, basic leucine zipper transcription factor 1 \| \|  \| LDOC1 \| Leucine zipper, down-regulated in cancer 1 \| \|  \| MATR3 \| Matrin 3 \| \|  \| TM6SF1 \| Transmembrane 6 superfamily member 1 \| \|  \| AGTR1 \| Angiotensin II receptor, type 1 \| \|  \| RHOA \| Ras homolog gene family, member A \| \|  \| ETS1 \| V-ets erythroblastosis virus E26 oncogene homolog 1 (avian) \| \|  \| MEIS1 \| Meis homeobox 1 \| \|  \| MAP3K7IP2 \| Mitogen-activated protein kinase kinase kinase 7 interacting protein 2 \| \|  \| CYR61 \| Cysteine-rich, angiogenic inducer, 61 \| \|  \| MyD88 \| Myeloid differentiation primary response gene (88) \| \|  \| FOXO3 \| Forkhead box O3 \| \|  \| SOCS1 \| Suppressor of cytokine signaling 1 \| \|  \| CCND1 \| Cyclin D1 \| \|  \| IKBKE \| Inhibitor of kappa light polypeptide gene enhancer in B-cells, kinase epsilon \| \|  \| KGF \|  \| \|  \| SPI1 \| Spleen focus forming virus (SFFV) proviral integration oncogene spi1 \| \|  \| FADD \| Fas (TNFRSF6)-associated via death domain \| \|  \| JARID2 \| Jumonji, AT rich interactive domain 2 \| \|  \| MeCP2 \| Methyl CpG binding protein 2 (Rett syndrome) \| \|  \| CEBPB \| CCAAT/enhancer binding protein (C/EBP), beta \| \|  \| SHIP \|  \| \|  \| SMAD2 \| SMAD family member 2 \| \|  \|  \|  \| \| miR-128 \| E2F3 \| E2F transcription factor 3 \| \|  \| NTRK3 \| Neurotrophic tyrosine kinase, receptor, type 3 \| \|  \| BMI1 \| BMI1 polycomb ring finger oncogene \| \|  \| ADORA2B \| Adenosine A2b receptor \| \|  \| DBI \| Diazepam binding inhibitor (GABA receptor modulator, acyl-Coenzyme A binding protein) \| \|  \| AGO1 \| Argonaute 1 \| \|  \| LDLR \| Low density lipoprotein receptor (familial hypercholesterolemia) \| \|  \| DCX \| Doublecortex; lissencephaly, X-linked (doublecortin) \| \|  \| RELN \| Reelin \| \|  \| MLL \| Myeloid/lymphoid or mixed-lineage leukemia (trithorax homolog, Drosophila) \| \|  \| AF4 \|  \| \|  \| TXNIP \| Thioredoxin interacting protein \| \|  \|  \|  \| \| miR-374b \| none \|  \| \|  \|  \|  \| \| miR-21 \| TPM1 \| Tropomyosin 1 (alpha) \| \|  \| PDCD4 \| Programmed cell death 4 (neoplastic transformation inhibitor) \| \|  \| CDKN1A \| Cyclin-dependent kinase inhibitor 1A (p21, Cip1) \| \|  \| FAS \| Fas (TNF receptor superfamily, member 6) \| \|  \| FAM3C \| Family with sequence similarity 3, member C \| \|  \| HIPK3 \| Homeodomain interacting protein kinase 3 \| \|  \| PRRG4 \| Proline rich Gla (G-carboxyglutamic acid) 4 (transmembrane) \| \|  \| ACTA2 \| Actin, alpha 2, smooth muscle, aorta \| \|  \| BTG2 \| BTG family, member 2 \| \|  \| BMPRII \|  \| \|  \| SESN1 \| Sestrin 1 \| \|  \| IL-6R \|  \| \|  \| SOCS5 \| Suppressor of cytokine signaling 5 \| \|  \| Glcci1 \| Glucocorticoid induced transcript 1 \| \|  \| APAF1 \| Apoptotic peptidase activating factor 1 \| \|  \| SLC16A10 \| Solute carrier family 16, member 10 (aromatic amino acid transporter) \| \|  \| SGK3 \| Serum/glucocorticoid regulated kinase family, member 3 \| \|  \| RP2 \| Retinitis pigmentosa 2 (X-linked recessive) \| \|  \| CDK6 \| Cyclin-dependent kinase 6 \| \|  \| CFL2 \| Cofilin 2 (muscle) \| \|  \| PDCD4 \| Programmed cell death 4 (neoplastic transformation inhibitor) \| \|  \| SERPINB5 \| Serpin peptidase inhibitor, clade B (ovalbumin), member 5 \| \|  \| PDCD4 \| Programmed cell death 4 (neoplastic transformation inhibitor) \| \|  \| NFIB \| Nuclear factor I/B \| \|  \| TIMP3 \| TIMP metallopeptidase inhibitor 3 (Sorsby fundus dystrophy, pseudoinflammatory) \| \|  \| RECK \| Reversion-inducing-cysteine-rich protein with kazal motifs \| \|  \| MTAP \| Methylthioadenosine phosphorylase \| \|  \| PDCD4 \| Programmed cell death 4 (neoplastic transformation inhibitor) \| \|  \| SOX5 \| SRY (sex determining region Y)-box 5 \| \|  \| MARCKS \| Myristoylated alanine-rich protein kinase C substrate \| \|  \| JAG1 \| Jagged 1 (Alagille syndrome) \| \|  \| BTG2 \| BTG family, member 2 \| \|  \| LRRFIP1 \| Leucine rich repeat (in FLII) interacting protein 1 \| \|  \| BMPR2 \| Bone morphogenetic protein receptor, type II (serine/threonine kinase) \| \|  \| CDC25A \| Cell division cycle 25 homolog A (S. pombe) \| \|  \| PTEN \| Phosphatase and tensin homolog (mutated in multiple advanced cancers 1) \| \|  \| E2F1 \| E2F transcription factor 1 \| \|  \| TGFBR2 \| Transforming growth factor, beta receptor II (70/80kDa) \| \|  \|  \|  \| \| miR-100 \| PLK1 \| Polo-like kinase 1 (Drosophila) \| \|  \| FGFR3 \| Fibroblast growth factor receptor 3 (achondroplasia, thanatophoric dwarfism) \| \|  \| IGF1R \| Insulin-like growth factor 1 receptor \| \|  \| raptor \|  \| \|  \|  \|  \| \| miR-125b \| SMO \| Smoothened homolog (Drosophila) \| \|  \| BMF \| Bcl2 modifying factor \| \|  \| BMPR1B \| Bone morphogenetic protein receptor, type IB \| \|  \| HuR \|  \| \|  \| CDK6 \| Cyclin-dependent kinase 6 \| \|  \| CDC25A \| Cell division cycle 25 homolog A (S. pombe) \| \|  \| KRT \|  \| \|  \| ST18 \| Suppression of tumorigenicity 18 (breast carcinoma) (zinc finger protein) \| \|  \| DICER1 \| Dicer1, Dcr-1 homolog (Drosophila) \| \|  \| CDKN2A \| Cyclin-dependent kinase inhibitor 2A (melanoma, p16, inhibits CDK4) \| \|  \|  \|  \| \| miR-140-3p \| none \|  \| \|  \|  \|  \| \| miR-125a-5p \| Lin28 \| Lin-28 homolog (C. elegans) \| \|  \| ERBB3 \| V-erb-b2 erythroblastic leukemia viral oncogene homolog 3 (avian) \| \|  \| p53 \|  \| \|  \| HuR \|  \| \|  \| ARID3B \| AT rich interactive domain 3B (BRIGHT-like) \| \|  \|  \|  \| \| miR-92a \| BMPRII \|  \| \|  \| ITGA5 \| Integrin, alpha 5 (fibronectin receptor, alpha polypeptide) \| \|  \|  \|  \| \| let-7c \| HMGA2 \| High mobility group AT-hook 2 \| \|  \| c-MYC \|  \| \|  \| TRIM71 \| Tripartite motif-containing 71 \| \|  \| HMGA2a \|  \| \|  \| TGFBR1 \| Transforming growth factor, beta receptor I (activin A receptor type II-like kinase, 53kDa) \| \|  \| EIF3S1 \|  \| \|  \| MED28 \| Mediator complex subunit 28 \| \|  \| RTCD1 \| RNA terminal phosphate cyclase domain 1 \| \|  \|  \|  \| \| miR-181b \| Tcl1 \|  \| \|  \| VSNL1 \| Visinin-like 1 \| \|  \| GRIA2 \| Glutamate receptor, ionotropic, AMPA 2 \| \|  \| CDX2 \| Caudal type homeobox 2 \| \|  \| GATA6 \| GATA binding protein 6 \| \|  \| NLK \| Nemo-like kinase \| \|  \| ESR1 \| Estrogen receptor 1 \| \|  \| PLAG1 \| Pleiomorphic adenoma gene 1 \| \|  \|  \|  \| \| let-7d \| DICER1 \| Dicer1, Dcr-1 homolog (Drosophila) \| \|  \| DAD3R \|  \| \|  \| DAD1 \| Defender against cell death 1 \| \|  \| EIF4G2 \| Eukaryotic translation initiation factor 4 gamma, 2 \| \|  \| RABGAP1L \| RAB GTPase activating protein 1-like \| \|  \| SMOX \| Spermine oxidase \| \|  \|  \|  \| \| miR-30c \| Ubc9 \|  \| \|  \|  \|  \| \| miR-27b \| CYP1B1 \| Cytochrome P450, family 1, subfamily B, polypeptide 1 \| \|  \| ST14 \| Suppression of tumorigenicity 14 (colon carcinoma) \| \|  \| PPARG \| Peroxisome proliferator-activated receptor gamma \| \|  \| MMP-13 \|  \| \|  \|  \|  \| \| miR-103 \| FBXW1B \|  \| \|  \| serbp1 \| SERPINE1 mRNA binding protein 1 \| \|  \| ICOS \| Inducible T-cell co-stimulator \| \|  \|  \|  \| \| miR-30a \| KRT7 \| Keratin 7 \| \|  \| beclin 1 \|  \| \|  \| TNRC6A \| Trinucleotide repeat containing 6A \| \|  \|  \|  \| \| miR-424 \| NFI-A \|  \| \|  \| PLAG1 \| Pleiomorphic adenoma gene 1 \| \|  \| FGFR1 \| Fibroblast growth factor receptor 1 (fms-related tyrosine kinase 2, Pfeiffer syndrome) \| \|  \| MEK1 \|  \| \|  \| Galectin-3 \|  \| \|  \|  \|  \| \| miR-22 \| PTEN \| Phosphatase and tensin homolog (mutated in multiple advanced cancers 1) \| \|  \| ESR1 \| Estrogen receptor 1 \| \|  \| PPARA \| Peroxisome proliferator-activated receptor alpha \| \|  \| BMP7 \| Bone morphogenetic protein 7 (osteogenic protein 1) \| \|  \| MAX \| MYC associated factor X \| \|  \|  \|  \| \| miR-186 \| FOXO1 \| Forkhead box O1 \| \|  \|  \|  \| \| miR-29a \| DNMT3B \| DNA (cytosine-5-)-methyltransferase 3 beta \| \|  \| SPARC \| Secreted protein, acidic, cysteine-rich (osteonectin) \| \|  \| BACE1 \| Beta-site APP-cleaving enzyme 1 \| \|  \| PIK3R1 \| Phosphoinositide-3-kinase, regulatory subunit 1 (p85 alpha) \| \|  \| CDC42 \| Cell division cycle 42 (GTP binding protein, 25kDa) \| \|  \| B7-H3 \|  \| \|  \| MCL-1 \|  \| \|  \| CXXC6 \| CXXC finger 6 \| \|  \| CDK6 \| Cyclin-dependent kinase 6 \| \|  \| COL3A1 \| Collagen, type III, alpha 1 (Ehlers-Danlos syndrome type IV, autosomal dominant) \| \|  \|  \|  \| \| miR-126 \| VCAM-1 \|  \| \|  \| Crk II \|  \| \|  \| p85beta \|  \| \|  \| IRS-1 \|  \| \|  \| VEGF-A \|  \| \|  \| TOM1 \| Target of myb1 (chicken) \| \|  \| prostein \|  \| \|  \|  \|  \| \| miR-27a \| prohibitin \|  \| \|  \| RUNX1 \| Runt-related transcription factor 1 (acute myeloid leukemia 1; aml1 oncogene) \| \|  \| GCA \| Grancalcin, EF-hand calcium binding protein \| \|  \| PEX7 \| Peroxisomal biogenesis factor 7 \| \|  \| FADD \| Fas (TNFRSF6)-associated via death domain \| \|  \| FOXO1 \| Forkhead box O1 \| \|  \|  \|  \| \| miR-20a \| E2F1 \| E2F transcription factor 1 \| \|  \| AML1 \|  \| \|  \| cyclin D1 \|  \| \|  \| BMPRII \|  \| \|  \| BCL2 \| B-cell CLL/lymphoma 2 \| \|  \| MEF2D \| Myocyte enhancer factor 2D \| \|  \| MAP3K12 \| Mitogen-activated protein kinase kinase kinase 12 \| \|  \| JAK1 \| Janus kinase 1 (a protein tyrosine kinase) \| \|  \| BIM \|  \| \|  \| IL-8 \|  \| \|  \|  \|  \| \| miR-143 \| ERK5 \|  \| \|  \| KRAS \| V-Ki-ras2 Kirsten rat sarcoma viral oncogene homolog \| \|  \| ERK5 \|  \| \|  \| FNDC3b \| Fibronectin type III domain containing 3B \| \|  \| DNMT3A \| DNA (cytosine-5-)-methyltransferase 3 alpha \| \|  \|  \|  \| \| miR-223 \| NFI-A \|  \| \|  \| STMN1 \| Stathmin 1/oncoprotein 18 \| \|  \| LMO2 \| LIM domain only 2 (rhombotin-like 1) \| \|  \| IRS-1 \|  \| \|  \| RhoB \| Ras homolog gene family, member B \| \|  \|  \|  \| \| miR-17 \| AIB1 \|  \| \|  \| AML1 \|  \| \|  \| VEGF \|  \| \|  \| cyclin D1 \|  \| \|  \| BMPRII \|  \| \|  \| BCL2 \| B-cell CLL/lymphoma 2 \| \|  \| MEF2D \| Myocyte enhancer factor 2D \| \|  \| MAP3K12 \| Mitogen-activated protein kinase kinase kinase 12 \| \|  \| Vim \| Vimentin \| \|  \| JAK1 \| Janus kinase 1 (a protein tyrosine kinase) \| \|  \| CDKN1A \| Cyclin-dependent kinase inhibitor 1A (p21, Cip1) \| \|  \| EDG1 \| Endothelial differentiation, sphingolipid G-protein-coupled receptor, 1 \| \|  \| BIM \|  \| \|  \| IL-8 \|  \| \|  \|  \|  \| \| miR-106b \| ITCH \| Itchy homolog E3 ubiquitin protein ligase (mouse) \| \|  \| CDKN1A \| Cyclin-dependent kinase inhibitor 1A (p21, Cip1) \| \|  \| E2F1 \| E2F transcription factor 1 \| \|  \|  \|  \| \| miR-96 \| Adcy6 \| Adenylate cyclase 6 \| \|  \| Mitf \| Microphthalmia-associated transcription factor \| \|  \| HTR1B \| 5-hydroxytryptamine (serotonin) receptor 1B \| \|  \| AQP5 \| Aquaporin 5 \| \|  \| CELSR2 \| Cadherin, EGF LAG seven-pass G-type receptor 2 (flamingo homolog, Drosophila) \| \|  \| ODF2 \| Outer dense fiber of sperm tails 2 \| \|  \| MYRIP \| Myosin VIIA and Rab interacting protein \| \|  \| RYK \| RYK receptor-like tyrosine kinase \| \|  \| IRS-1 \|  \| \|  \| FOXO1 \| Forkhead box O1 \| \|  \|  \|  \| \| miR-140-5p \| VEGF \|  \| \|  \| HDAC4 \| Histone deacetylase 4 \| \|  \| IGFBP-5 \|  \| \|  \|  \|  \| \| miR-15a \| DMTF1 \| Cyclin D binding myb-like transcription factor 1 \| \|  \| BCL2 \| B-cell CLL/lymphoma 2 \| \|  \| VEGF \|  \| \|  \| PDCD4 \| Programmed cell death 4 (neoplastic transformation inhibitor) \| \|  \| RAB21 \| RAB21, member RAS oncogene family \| \|  \| IGSF4 \|  \| \|  \| SCAP2 \|  \| \|  \| Rab9B \| RAB9B, member RAS oncogene family \| \|  \| Tpi1 \| Triosephosphate isomerase 1 \| \|  \| c-Myb \|  \| \|  \| CCND1 \| Cyclin D1 \| \|  \| BMI-1 \|  \| \|  \|  \|  \| \| miR-30b \| none \|  \| \|  \|  \|  \| \| miR-130a \| MCSF \|  \| \|  \| MAFB \| V-maf musculoaponeurotic fibrosarcoma oncogene homolog B (avian) \| \|  \| GAX \|  \| \|  \| HOXA5 \| Homeobox A5 \| \|  \|  \|  \| \| miR-222 \| KIT \| V-kit Hardy-Zuckerman 4 feline sarcoma viral oncogene homolog \| \|  \| p27Kip1 \|  \| \|  \| p27 \|  \| \|  \| p27Kip1 \|  \| \|  \| ER alpha \|  \| \|  \| BIM \|  \| \|  \| MMP1 \| Matrix metallopeptidase 1 (interstitial collagenase) \| \|  \| SOD2 \| Superoxide dismutase 2, mitochondrial \| \|  \| CDKN1C \| Cyclin-dependent kinase inhibitor 1C (p57, Kip2) \| \|  \| PPP2R2A \| Protein phosphatase 2 (formerly 2A), regulatory subunit B, alpha isoform \| \|  \| c-fos \|  \| \|  \|  \|  \| \| miR-30e \| UBE2I \| Ubiquitin-conjugating enzyme E2I (UBC9 homolog, yeast) \| \|  \|  \|  \| \| miR-29c \| DNMT3B \| DNA (cytosine-5-)-methyltransferase 3 beta \| \|  \| COL3A1 \| Collagen, type III, alpha 1 (Ehlers-Danlos syndrome type IV, autosomal dominant) \| \|  \| COL4A1 \| Collagen, type IV, alpha 1 \| \|  \| COL15A1 \| Collagen, type XV, alpha 1 \| \|  \| LAMC1 \| Laminin, gamma 1 (formerly LAMB2) \| \|  \| TDG \| Thymine-DNA glycosylase \| \|  \| FUSIP1 \| FUS interacting protein (serine/arginine-rich) 1 \| \|  \| COL1A1 \| Collagen, type I, alpha 1 \| \|  \| COL1A2 \| Collagen, type I, alpha 2 \| \|  \| COL4A2 \| Collagen, type IV, alpha 2 \| \|  \| fibrillin 1 \|  \| \|  \| CDC42 \| Cell division cycle 42 (GTP binding protein, 25kDa) \| \|  \| PIK3R1 \| Phosphoinositide-3-kinase, regulatory subunit 1 (p85 alpha) \| \|  \|  \|  \| \| miR-18a \| ESR1 \| Estrogen receptor 1 \| \|  \| thbs1 \| Thrombospondin 1 \| \|  \| BIM \|  \| \|  \| CTGF \| Connective tissue growth factor \| \|  \|  \|  \| \| miR-29b \| Tcl1 \|  \| \|  \| MCL1 \| Myeloid cell leukemia sequence 1 (BCL2-related) \| \|  \| DNMT3A \| DNA (cytosine-5-)-methyltransferase 3 alpha \| \|  \| DNMT3B \| DNA (cytosine-5-)-methyltransferase 3 beta \| \|  \| BACE1 \| Beta-site APP-cleaving enzyme 1 \| \|  \| CDC42 \| Cell division cycle 42 (GTP binding protein, 25kDa) \| \|  \| PIK3R1 \| Phosphoinositide-3-kinase, regulatory subunit 1 (p85 alpha) \| \|  \| SP1 \| Sp1 transcription factor \| \|  \| MCL-1 \|  \| \|  \| CXXC6 \| CXXC finger 6 \| \|  \| CDK6 \| Cyclin-dependent kinase 6 \| \|  \| COL1A1 \| Collagen, type I, alpha 1 \| \|  \| SP1 \| Sp1 transcription factor \| \|  \| COL3A1 \| Collagen, type III, alpha 1 (Ehlers-Danlos syndrome type IV, autosomal dominant) \| \|  \|  \|  \| \| miR-142-5p \| none \|  \| \|  \|  \|  \| \| miR-144 \| none \|  \| \|  \|  \|  \| \| miR-423-3p \| none \|  \| \|  \|  \|  \| \| miR-142-3p \| none \|  \| \|  \|  \|  \| \| miR-19b \| ESR1 \| Estrogen receptor 1 \| \|  \| CTGF \| Connective tissue growth factor \| \|  \|  \|  \| \| miR-19a \| PTEN \| Phosphatase and tensin homolog (mutated in multiple advanced cancers 1) \| \|  \| BMPRII \|  \| \|  \| NR4A2 \| Nuclear receptor subfamily 4, group A, member 2 \| \|  \| ERBB4 \| V-erb-a erythroblastic leukemia viral oncogene homolog 4 (avian) \| \|  \| ESR1 \| Estrogen receptor 1 \| \|  \| CCND1 \| Cyclin D1 \| \|  \| thbs1 \| Thrombospondin 1 \| \|  \| BIM \|  \| \|  \| CTGF \| Connective tissue growth factor \| \|  \|  \|  \| \| miR-32 \| none \|  \| \|  \|  \|  \| \| miR-101 \| N-MYC \|  \| \|  \| ICOS \| Inducible T-cell co-stimulator \| \|  \| EZH2 \| Enhancer of zeste homolog 2 (Drosophila) \| \|  \| Cox-2 \|  \| \|  \| MCL1 \| Myeloid cell leukemia sequence 1 (BCL2-related) \| \|  \|  \|  \| \| miR-141 \| Clock \| Clock homolog (mouse) \| \|  \| TGF beta2 \|  \| \|  \| SIP1 \| Survival of motor neuron protein interacting protein 1 \| \|  \| MKK4 \|  \| \|  \| ERBB2IP \| Erbb2 interacting protein \| \|  \| BAP1 \| BRCA1 associated protein-1 (ubiquitin carboxy-terminal hydrolase) \| \|  \| KLHL20 \| Kelch-like 20 (Drosophila) \| \|  \| PTPRD \| Protein tyrosine phosphatase, receptor type, D \| \|  \| ELMO2 \| Engulfment and cell motility 2 \| \|  \| WDR37 \| WD repeat domain 37 \| |  |  |  |  |  | PPP2R2A | Protein phosphatase 2 (formerly 2A), regulatory subunit B, alpha isoform |
| --- | --- | --- | --- | --- | --- | --- | --- | --- | --- | --- | --- | --- | --- | --- | --- | --- | --- | --- | --- | --- | --- | --- | --- | --- | --- | --- | --- | --- | --- | --- | --- | --- | --- | --- | --- | --- | --- | --- | --- | --- | --- | --- | --- | --- | --- | --- | --- | --- | --- | --- | --- | --- | --- | --- | --- | --- | --- | --- | --- | --- | --- | --- | --- | --- | --- | --- | --- | --- | --- | --- | --- | --- | --- | --- | --- | --- | --- | --- | --- | --- | --- | --- | --- | --- | --- | --- | --- | --- | --- | --- | --- | --- | --- | --- | --- | --- | --- | --- | --- | --- | --- | --- | --- | --- | --- | --- | --- | --- | --- | --- | --- | --- | --- | --- | --- | --- | --- | --- | --- | --- | --- | --- | --- | --- | --- | --- | --- | --- | --- | --- | --- | --- | --- | --- | --- | --- | --- | --- | --- | --- | --- | --- | --- | --- | --- | --- | --- | --- | --- | --- | --- | --- | --- | --- | --- | --- | --- | --- | --- | --- | --- | --- | --- | --- | --- | --- | --- | --- | --- | --- | --- | --- | --- | --- | --- | --- | --- | --- | --- | --- | --- | --- | --- | --- | --- | --- | --- | --- | --- | --- | --- | --- | --- | --- | --- | --- | --- | --- | --- | --- | --- | --- | --- | --- | --- | --- | --- | --- | --- | --- | --- | --- | --- | --- | --- | --- | --- | --- | --- | --- | --- | --- | --- | --- | --- | --- | --- | --- | --- | --- | --- | --- | --- | --- | --- | --- | --- | --- | --- | --- | --- | --- | --- | --- | --- | --- | --- | --- | --- | --- | --- | --- | --- | --- | --- | --- | --- | --- | --- | --- | --- | --- | --- | --- | --- | --- | --- | --- | --- | --- | --- | --- | --- | --- | --- | --- | --- | --- | --- | --- | --- | --- | --- | --- | --- | --- | --- | --- | --- | --- | --- | --- | --- | --- | --- | --- | --- | --- | --- | --- | --- | --- | --- | --- | --- | --- | --- | --- | --- | --- | --- | --- | --- | --- | --- | --- | --- | --- | --- | --- | --- | --- | --- | --- | --- | --- | --- | --- | --- | --- | --- | --- | --- | --- | --- | --- | --- | --- | --- | --- | --- | --- | --- | --- | --- | --- | --- | --- | --- | --- | --- | --- | --- | --- | --- | --- | --- | --- | --- | --- | --- | --- | --- | --- | --- | --- | --- | --- | --- | --- | --- | --- | --- | --- | --- | --- | --- | --- | --- | --- | --- | --- | --- | --- | --- | --- | --- | --- | --- | --- | --- | --- | --- | --- | --- | --- | --- | --- | --- | --- | --- | --- | --- | --- | --- | --- | --- | --- | --- | --- | --- | --- | --- | --- | --- | --- | --- | --- | --- | --- | --- | --- | --- | --- | --- | --- | --- | --- | --- | --- | --- | --- | --- | --- | --- | --- | --- | --- | --- | --- | --- | --- | --- | --- | --- | --- | --- | --- | --- | --- | --- | --- | --- | --- | --- | --- | --- | --- | --- | --- | --- | --- | --- | --- | --- | --- | --- | --- | --- | --- | --- | --- | --- | --- | --- | --- | --- | --- | --- | --- | --- | --- | --- | --- | --- | --- | --- | --- | --- | --- | --- | --- | --- | --- | --- | --- | --- | --- | --- | --- | --- | --- | --- | --- | --- | --- | --- | --- | --- | --- | --- | --- | --- | --- | --- | --- | --- | --- | --- | --- | --- | --- | --- | --- | --- | --- | --- | --- | --- | --- | --- | --- | --- | --- | --- | --- | --- | --- | --- | --- | --- | --- | --- | --- | --- | --- | --- | --- | --- | --- | --- | --- | --- | --- | --- | --- | --- | --- | --- | --- | --- | --- | --- | --- | --- | --- | --- | --- | --- | --- | --- | --- | --- | --- | --- | --- | --- | --- | --- | --- | --- | --- | --- | --- | --- | --- | --- | --- | --- | --- | --- | --- | --- | --- | --- | --- | --- | --- | --- | --- | --- | --- | --- | --- | --- | --- | --- | --- | --- | --- | --- | --- | --- | --- | --- | --- | --- | --- | --- | --- | --- | --- | --- | --- | --- | --- | --- | --- | --- | --- | --- | --- | --- | --- | --- | --- | --- | --- | --- | --- | --- | --- | --- | --- | --- | --- | --- | --- | --- | --- | --- | --- | --- | --- | --- | --- | --- | --- | --- | --- | --- | --- | --- | --- | --- | --- | --- | --- | --- | --- | --- | --- | --- | --- | --- | --- | --- | --- | --- | --- | --- | --- | --- | --- | --- | --- | --- | --- | --- | --- | --- | --- | --- | --- | --- | --- | --- | --- | --- | --- | --- | --- | --- | --- | --- | --- | --- | --- | --- | --- | --- | --- | --- | --- | --- | --- | --- | --- | --- | --- | --- | --- | --- | --- | --- | --- | --- | --- | --- | --- | --- | --- | --- | --- | --- | --- | --- | --- | --- | --- | --- | --- | --- | --- | --- | --- | --- | --- | --- | --- | --- | --- | --- | --- | --- | --- | --- | --- | --- | --- | --- | --- | --- | --- | --- | --- | --- | --- | --- | --- | --- | --- | --- | --- | --- | --- | --- | --- | --- | --- | --- | --- | --- | --- | --- | --- | --- | --- | --- | --- | --- | --- | --- | --- | --- | --- | --- | --- | --- | --- | --- | --- | --- | --- | --- | --- | --- | --- | --- | --- | --- | --- | --- | --- | --- | --- | --- | --- | --- | --- | --- | --- | --- | --- | --- | --- | --- | --- | --- | --- | --- | --- | --- | --- | --- | --- | --- | --- | --- | --- | --- | --- | --- | --- | --- | --- | --- | --- | --- | --- | --- | --- | --- | --- | --- | --- | --- | --- | --- | --- | --- | --- | --- | --- | --- | --- | --- | --- | --- | --- | --- | --- | --- | --- | --- | --- | --- | --- | --- | --- | --- | --- | --- | --- | --- | --- | --- | --- | --- | --- | --- | --- | --- | --- | --- | --- | --- | --- | --- | --- | --- | --- | --- | --- | --- | --- | --- | --- | --- | --- | --- | --- | --- | --- | --- | --- | --- | --- | --- | --- | --- | --- | --- | --- | --- | --- | --- | --- | --- | --- | --- | --- | --- | --- | --- | --- | --- | --- | --- | --- | --- | --- | --- | --- | --- | --- | --- | --- | --- | --- | --- | --- | --- | --- | --- | --- | --- | --- | --- | --- | --- | --- | --- | --- | --- | --- | --- | --- | --- | --- | --- | --- | --- | --- | --- | --- | --- | --- | --- | --- | --- | --- | --- | --- | --- | --- | --- | --- | --- | --- | --- | --- | --- | --- | --- | --- | --- | --- | --- | --- | --- | --- | --- | --- | --- | --- | --- | --- | --- | --- | --- | --- | --- | --- | --- | --- | --- | --- | --- | --- | --- | --- | --- | --- | --- | --- | --- | --- | --- | --- | --- | --- | --- | --- | --- | --- | --- | --- | --- | --- | --- | --- | --- | --- | --- | --- | --- | --- | --- | --- | --- | --- | --- | --- | --- | --- | --- | --- | --- | --- | --- | --- | --- | --- | --- | --- | --- | --- | --- | --- | --- | --- |
